# Oral TNFα Modulation Alters Neutrophil Infiltration, Improves Cognition and Diminishes Tau and Amyloid Pathology in the 3xTgAD Mouse Model

**DOI:** 10.1371/journal.pone.0137305

**Published:** 2015-10-05

**Authors:** S. Prasad Gabbita, Ming F. Johnson, Naomi Kobritz, Pirooz Eslami, Aleksandra Poteshkina, Sridhar Varadarajan, John Turman, Frank Zemlan, Marni E. Harris-White

**Affiliations:** 1 P2D Bioscience, Inc., Cincinnati, Ohio, United States of America; 2 University of North Carolina Wilmington, Department of Chemistry and Biochemistry, Wilmington, North Carolina, United States of America; 3 Veterans Administration-Greater Los Angeles Healthcare System, Los Angeles, California, United States of America; 4 University of California Los Angeles, David Geffen School of Medicine, Los Angeles, California, United States of America; Boston University Goldman School of Dental Medicine, UNITED STATES

## Abstract

Cytokines such as TNFα can polarize microglia/macrophages into different neuroinflammatory types. Skewing of the phenotype towards a cytotoxic state is thought to impair phagocytosis and has been described in Alzheimer’s Disease (AD). Neuroinflammation can be perpetuated by a cycle of increasing cytokine production and maintenance of a polarized activation state that contributes to AD progression. In this study, 3xTgAD mice, age 6 months, were treated orally with 3 doses of the TNFα modulating compound isoindolin-1,3 dithione (IDT) for 10 months. We demonstrate that IDT is a TNFα modulating compound both *in vitro* and *in vivo*. Following long-term IDT administration, mice were assessed for learning & memory and tissue and serum were collected for analysis. Results demonstrate that IDT is safe for long-term treatment and significantly improves learning and memory in the 3xTgAD mouse model. IDT significantly reduced paired helical filament tau and fibrillar amyloid accumulation. Flow cytometry of brain cell populations revealed that IDT increased the infiltrating neutrophil population while reducing TNFα expression in this population. IDT is a safe and effective TNFα and innate immune system modulator. Thus small molecule, orally bioavailable modulators are promising therapeutics for Alzheimer’s disease.

## Introduction

Neuroinflammation is now widely recognized as a primary feature of Alzheimer’s Disease (AD) pathology. Alois Alzheimer himself recognized that microglia, the brain’s resident immune cells, were found to form a halo around senile plaques. Long underappreciated as the brain’s ‘glue’, it would be decades before we began to understand the complexities of the immune system in the brain and that neuroinflammation is an important therapeutic target for AD.

In addition to brain-resident microglia, the central nervous system is also engaged with the peripheral immune system. Early studies demonstrate that peripheral immune cells flow into and out of the central nervous system [1,2] and that these cells work as surveillance agents under normal conditions and are present in low numbers. Disease conditions and the concomitant rise in cytokines are known to orchestrate an increase in leukocyte extravasation and accumulation in the CNS. Studies in AD patients [3,4] and in AD animal models [5–7] provide evidence for increased trafficking of leukocytes into the brain and interaction with brain resident microglia. Despite general consensus on this increased trafficking, it remains unclear whether this increased infiltration is detrimental to or beneficial for the AD brain. Although not systematically studied in the human AD brain, some animal model studies demonstrate that increased inflammation can reduce amyloid pathology [8]. However, modulation of macrophage signaling pathways can also attenuate amyloid deposits [9]. The precise relationship between AD pathology and the innate immune response remains to be clearly defined.

The status of microglia/macrophage activity can be graded along a spectrum [10]. The classic pro-inflammatory state is dominated by expression of cytokines such as TNFα and IL-1β. At the other end of the spectrum is a state considered alternatively activated and is thought to shift the phenotype towards enhanced clearance/phagocytosis, tissue regeneration and renewal [11]. The plasticity and evolution of microglia/macrophage status may be what is hindered during disease processes and therapeutics designed to harness innate immune cell responses may be effective interventions for CNS pathologies such as AD. Comprehensive investigations of the spectrum of phenotypes and efficacy metrics will help to define standards and allow the interpretation of innate cell status and contribution to disease state.

The purpose of this study was to determine if long-term modulation of TNFα, a cytokine heavily implicated in AD [12], was safe and effective in tuning the innate immune system and reducing AD pathology and cognitive impairment. We found that a small molecule TNFα modulator, delivered orally for 10 months in the 3xTgAD mouse model, was safe and without adverse side effects. Paired helical filament (PHF) tau and fibrillar amyloid were reduced while cognitive abilities were improved. These improvements may be the result of transformations to the innate immune system, primarily CNS neutrophilia and improved clearance of amyloid and tau pathology by microglia/macrophages.

## Methods

### Animals

Euthanasia was conducted as part of this study. The method was by Pentobarbital overdose followed by cardiac perfusion and decapitation. All experiments were approved by the institutional animal care and use committee (protocol #08028–12) at the Veterans Administration-Greater Los Angeles Healthcare System (Animal welfare assurance #3002–01) in accordance with National Institutes of Health guidelines.

C57/Bl6 male mice were used for LPS injection studies (LPS serotype O55:B5 from E. Coli). IDT was prepared at 10 mg/mL as a suspension in a vehicle of 0.2% Tween80 and 0.5% methylcellulose in sterile water. IDT at a dose of 50 mg/kg (gavage; 150 μL/30g mouse) was administered 30 minutes prior to a 5 mg/kg LPS injection (i.p.). Mice were monitored for signs of distress every 20 minutes throughout the 4 hour survival period. There were no animal deaths as a result of LPS and/or IDT treatment. At the 4 hour experimental endpoint, mice were humanely euthanized for blood and tissue collection.

Male and female homozygous3xTgAD mice expressing transgenic Swedish mutant human amyloid precursor protein (APPswe), transgenic mutant Tau^P301L^ and knock-in mutant presenilin-1 (PS1^M146V^) [13] were used for long-term oral dosing of IDT. 7 month-old (n = 14-16/group; equal gender distribution) mice were fed control or IDT-formulated diets, housed under a standard 12 h light:dark light cycle and given free access to water. IDT was formulated at 80, 200 and 400 mg/kg into AIN93G rodent diet (Dyets, Inc) and stored at 4°C until use. Animals were fed a defined amount of food per day (approx. 4-5g/day depending on animal weight) in order to achieve proper dosing (10, 25 or 50 mg/kg/day). The max dose of 50 mg/kg was chosen based on our premilinary in vivo LPS studies and previously published work with a similar drug (dithiothalidomide) [6] showing efficacy. IDT is more potent in vitro and we sought to determine if lower doses (10 and 25 mg/kg) would also be effective in the 3xTgAD mouse model. With rare exceptions, all mice consumed their food each day and no taste aversion to the IDT diet was evident. Male and female non-transgenic control mice were the same background strain as the 3xTgAD mice (C57BL6/129SVJ).

### Cmax and Brain/Plasma ratio

Brain and plasma IDT levels were assayed in male BalbC mice, age 8 weeks of age. IDT was dissolved in vehicle of 5% N-methyl-2-pyrrolidone (NMP), 5% DMSO, 10% Cremophor EL and 80% Mill-Q water. Mice were injected intraperitoneally with 25 mg/kg IDT. Brain and plasma samples were collected at 30 and 60 min post injection. Brain samples were weighed and homogenized by addition of 3x volumes of phosphate buffered saline (PBS). From 50 uL of brain homogenate or plasma, protein was precipitated by 0.2 mL 10% tetrahydrofuran in acetonitrile. IDT concentration was determined using LC-MS/MS using a 209 μg/mL IDT stock solution and quality control levels of 30.2, 1256 and 1822 ng/ml with 200 ng/mL tolbutamide as internal standard.

Following once daily oral gavage administration of IDT suspension (0.2% Tween80 and 0.5% methylcellulose in sterile water; n = 4/ time point) for 10 days, approximately 0.7 mL of blood was collected from the retro-orbital plexus sinus under isoflurane anesthesia and transferred into labeled tubes containing K_2_EDTA (100 mM per mL of blood) as anticoagulant. Blood samples were mixed by manual inversion 4 to 5 times and kept on ice until centrifugation at 5000 rpm for 5 minutes at 4°C. After separation, about 300 μL of plasma was diluted with 15 μL of 1N HCl and mixed gently, and stored at -70 ± 10°C until they were analyzed. The plasma samples were analyzed for quantification of IDT using LC-MS/MS detection with a lower limit of quantification of 5.05 ng/mL.

### Barnes Maze

The method used in our lab was adapted from Sharma et al [14] with some modification. A 20-hole Barnes maze apparatus was used (Any Maze, Stoelting, Wood Dale, IL). White curtains are used around the maze to reduce room cues. Three cues were placed in close proximity to the maze for use in spatial navigation (escape hole location and cues were changed for repeated testing). In the pre-training trial, the mouse is placed in the middle of the maze under a dark colored box allowing the mouse to be in random orientation before each trial. After 10 s had elapsed, the chamber was lifted, and the mouse allowed to explore the maze for 3 min. Aversive stimuli (sounds, wind, harsh lighting) were not utilized. Errors and latency were recorded during acquisition and testing. Errors are defined as nose pokes and head deflections over any hole that does not have the target box. Latency is defined as the time it takes to locate the target box. Mice are trained for four trials per day for 4 days with an inter-trial interval of at least 15 min. After each trial, the entire maze is cleaned with an unscented, mild, dilute soap solution. Mice not actively moving throughout the maze were eliminated from the analysis. 48 hrs after the last training trial, a probe trial was conducted to evaluate short-term memory retention. Any Maze software was used to capture and analyze the data.

### Nesting behavior

The nest-building task was administered as previously described [15,16] with slight modifications. Nest-building is dependent on step-by-step planning and organization, shown in many tests to be sensitive to lesions of prefrontal cortex (29). Mice were individually housed and given a cotton nestlet and equivalent numbers of corrugated brown paper strips and given 24 h to construct a nest. Cages were photographed and the presence and quality of nests scored by three independent raters on a scale from 0 to 5 with a 0 score being an untouched cotton nestlet and a 5 being a perfectly round, 4-walled nest that integrates both nesting materials and nearly conceals the mouse within.

### Tissue collection

At the end of behavior testing (16 months of age), animals were anesthetized with 100 mg/kg pentobarbital and a blood sample taken via cardiac puncture followed by cardiac perfusion with HEPES buffer (10 mM HEPES, 137 mM NaCl, 4.6 mM NaCl, 4.6 mM KCl, 1.1 mM KH_2_PO_4_, 0.6 mM MgSO_4_ and 1.1 mM EDTA, 1 mM Na_3_VO_4_ and protease inhibitor cocktail). Hippocampus and cortex were dissected from one hemisphere, snap frozen in N_2_(l) and stored at -80°C or stored at -20°C in RNALater (Ambion, Inc.). The contralateral hemisphere was immersion fixed in formalin (Fisher) for 24 hrs followed by step-wise (10-20-30%) sucrose cryopreservation and embedding in preparation for cryostat sectioning.

### Immunohistochemistry

Formalin fixed, cryopreserved brains were sagitally sectioned on a Leica cryostat at 50 μm thickness and stored at -20°C until immunolabeling. Immunohistochemical labeling was performed on free-floating sections using a VectaStain Elite ABC kit (Vector Laboratories) with diaminobenzadine chromagen. Antibodies were: 6E10 (Aβ1–16; 1:1000, Covance, Emeryville, CA); 12F4 (Specific to the C-terminus of Aβ specific for the isoform ending at the 42^nd^ amino acid; 1:1000, Covance); AT8 (phospho-Tau; 1:1000, Thermo Scientific); Iba-1 (microglia; 1:800, Wako). Sections were mounted on glass slides and coverslipped using Permount® mounting media (Fisher). Specificity of antibody immunoreactivity (ir) was confirmed by elimination of the primary antibody during the staining procedure. This negative control staining resulted in the loss of staining for these antibodies as predicted.

6E10, 12F4, AT8 and Iba-1 labeling intensities and counts were measured using Image Pro software (Media Cybernetics) or Microbrightfield software to quantify images of the subiculum, hippocampus and entorhinal cortex (as indicated in the figure legends) from five brain sections per mouse. Light intensity remained static during the acquisition of images for analysis and all images were treated identically.

### Thioflavin-S staining

Air-dried brain sections mounted onto glass slides were stained for thioflavin-S as previously described [17]. Briefly, slides were hydrated in ethanol (100/95/80%) followed by a 15-min incubation in a freshly made thioflavin-S solution (1% in 80% ethanol; 0.2 μm filtered), followed by ethanol (80/95/100%) and coverslipping. Thioflavin-s positive structures were visualized using fluorescent microscopy. Measurements in the subicular/CA1 region were performed on 5 brain sections/mouse using MBF software.

### Tissue preparation for enzyme-linked immunosorbent assays

In brief, Tissue extraction reagent (Invitrogen, Camarillo, CA Cat # FNN0071) containing protease and phosphatase inhibitors (Roche, Complete Ultra Mini # 05892970001; PhosphoSTOP #04906845001) was added to each tissue sample. Tissue was homogenized with 20 passes of a Teflon pestle homogenizer. For TNFα and tau ELISA, homogenates were centrifuged at 10,000 rpm for 10 min at 4°C and the resulting supernatants were removed and stored at -20°C until use. For amyloid ELISAs, samples were prepared as previously described [18]. Tissue homogenates were ultracentifuged (100,000 x g, 1 h, 4°C), and supernatants were recovered as the soluble fractions. Pellets containing insoluble material were mechanically dissociated in formic acid (70%) and ultracentrifuged (100,000 x g, 1 h, 4°C). Supernatants were kept as the insoluble fraction. Before analysis, formic acid fractions were neutralized to pH 7.5 with 1 M Tris-HCl, pH 10.8, containing 25 mM betaine (20 times dilution).

### ELISA for TNFα, Amyloid and Tau

The levels of TNFα in culture media or mouse cortical supernatants were measured using Siloam Biosciences Optimiser™ Microfluidic ELISA Plates. This technology combines microfluidics with 96-well architecture to offer dramatic improvements over conventional ELISA, such as 50 fold-reduced sample volume, 2 fold-reduced assay time, while providing ultra-high sensitivity and still using conventional ELISA reagents. Anti-mouse TNFα (capture), biotinylated anti-mouse TNFα (detection), recombinant mouse TNFα and Streptavidin-HRP were from R & D Systems (DY410). Standards ranged from 31.2 pg/mL to 2000 pg/mL in all assays. Samples were appropriately diluted to fall within the optimal range and not below, generally a 1:10–1:20 dilution.

Amyloid and tau ELISAs were performed using commercially available kits (Novex^TM^ by Life Technologies) following manufacturer’s instructions. Human amyloid beta 40 kit (KHB3481), amyloid beta 42 ultra sensitive kit (KHB3544), total tau kit (KHB0042) and tau [pSer396] kit (KHB7031). Sample dilutions were determined so that absorbance values fell within the limits of the standard curves.

### Real Time Quantitative PCR Analysis

Samples were stored in RNAlater (Ambion Inc., Austin, Texas) at -20°C. Total RNA was extracted using TRI reagent (Sigma, St. Louis, Missouri) and BCP (MRC, Cincinnati, Ohio) as a phase separation reagent. RNA was purified using the RNeasy Kit (Qiagen, Germantown, Maryland) and then treated with RNase-free DNase set (Qiagen). Purified RNA was quantified spectrophotometrically. RNA was reverse transcribed to complementary DNA (cDNA) using the RT^2^ First Strand Kit (Qiagen). 20 μL of cDNA for each sample was diluted with 91 μL of RNase free water for PCR array use. A custom-made, 96-well, real time PCR array (Qiagen RT^2^ Profiler CAMP12783; IL-6, NM_031168.1, Mm00446190_m1; IL-1β, NM_008361.3, Mm00434228_m1; IL-12a, NM_008351.2, Mm00434165_m1; IL-12b, NM_008352.2, Mm00434174_m1; ARG1, NM_007482.3, Mm00475988_m1; YM1 (Chil3), NM_009892.2, Mm00657889_mH; CD86, NM_019388.3, Mm00444543_m1; FcγR1, NM_010186.5, Mm00438874_m1; FcγR3, NM_010188.5, Mm00438882_m1; TGFβ1, NM_011577.1, Mm01178820_m1; SPHK1, NM_011451.3, Mm01252544_m1) was performed using an ABI 7300 Sequence Detection System (Applied Biosystems, Foster City, CA). Thermal cycling conditions included a holding stage at 95°C for 10 minutes, followed by 40 cycles of denaturation at 95°C for 15 seconds and annealing/primer extension at 60°C for one minute, then a final stage of 95°C (15 seconds), 60°C (1 minute) and 95°C (15 seconds). Each PCR array plate contained a proprietary panel of controls for genomic DNA contamination (GDC), a reverse transcription control (RTC) and a positive PCR control (PPC). All genes were normalized to glyceraldehyde-3-phosphate dehydrogenase (GAPDH, NM_008084, Mm.343110). Fold regulation was determined by the 2^–ΔΔCt^ method using the GeneGlobe Data Analysis Center.

### mRNA decay

We assessed the stability of TNFα mRNA in the presence of IDT using actinomycin D (ActD; 5 μg/mL), a well-known inhibitor of transcription [19]. BV2 cells were stimulated with 100 ng/mL LPS for 4 hrs before assessment of 25 μM IDT on mRNA stability. Following LPS removal, IDT and ActD were added and samples collected every 15 minutes. RNA from BV2 cells was extracted, purified and reverse transcribed to cDNA as described above. The amounts of mouse TNFα mRNA were determined by amplification of the cDNA target using the RT^2^ qPCR Primer Assay for TNFα (Qiagen). To normalize the quantification of TNFα mRNA for possible differences in the amount of each cDNA template, 18S rRNA served as a housekeeping gene. PCR amplifications of TNFα and 18S rRNA genes were carried out in conjunction with RT^2^ qPCR SYBR Green Master Mix (Qiagen). Each cDNA sample was tested in triplicate. The following temperature parameters were cycled for 40 times: 15 seconds at 95°C, 1 minute at 60°C. Standard curves were constructed for 18S rRNA as an internal standard and for TNFα gene. The amounts of TNFα mRNA gene expression were normalized by division by the amount of 18SrRNA mRNA. Relative gene expression levels correspond to fold induction (2^–ΔΔCt^) compared with untreated cells.

### Flow Cytometry

Dissociation of neural tissues to single-cell suspension was performed on a gentleMACS Octo Dissociator (Miltenyi Biotec, San Diego, CA). Mice were perfused with Hanks´ Balanced Salt Solution (HBSS) without Ca^2+^ and Mg^2+^ (Gibco). Whole brains (without cerebellum) were processed using the Neural Tissue Dissociation Kit (P) (Miltenyi Biotec) and run using the gently MACS NTDK Program for 22 min. At the end of the run, samples were centrifuged at r.t. for 1 minute at 300 x g, resuspended with 10 mL of HBSS with Ca^2+^ and Mg^2+^ (w) (Gibco) and passed through a 40 μm cell strainer. Cells were pelleted at 300 x g for 10 minutes and washed again with 10 mL HBSS. Cells were resuspended in 1.8 ml of AutoMACS Buffer (Miltenyi Biotec) and 200 μl of Myelin Removal Beads II (Miltenyi Biotec) and incubated for 15 minutes at 4°C, washed with 10X the labeling volume of buffer and centrifuged at 300 x g for 10 minutes. For magnetic separation, cells were passed through MACS LS Columns (Miltenyi Biotec) and placed on the MACS separator (Miltenyi Biotec). Each sample was resuspended in 3 ml of AutoMACS buffer and passed through 70 μm filters (Miltenyi Biotec) on LS Columns (1 mL for each column). Each column was washed twice with 1ml of AutoMACS buffer. Fractions were combined and cells were counted on a Countess automated cell counter (Invitrogen, Grand Islands, NY).

For preparation of spleen cells, whole spleens were placed onto a 70 μm cell strainer in a petridish containing 8 mL of DMEM-10 [(Gibco #21063–029) with 10% FBS (Hyclone #SH30071.03)]. Using the plunger end of the syringe, the spleen was dissociated. The cell strainer was rinsed with an additional 5 mL of media and the cell suspension centrifuged at 1200 rpm for 5 minutes. The pellet was resuspended in 1 mL ACK lysis buffer [0.15M NH_4_Cl, 1.0 M KHCO_3_, 0.1mM EDTA-Na_2_, pH 7.2], incubated at r.t. for 10 minutes and centrifuged with 9 mL DMEM-10. The pellet was resuspended in 10 mL DMEM-10, cell suspension passed through a 70 μm cell strainer and the cells counted on a Countess, automated cell counter. After the cells were counted, splenocytes were processed identically to the brain cells.

For staining, cells were centrifuged and incubated in GolgiStop (BD Bioscience) in phenol red-free complete RMPI (Gibco # 11835–030) with HIFBS (Gibco #10438–026) for 4 hours at 37°C (6 million cells/6 mL). After a 4 hr incubation, cells were combined into 5 mL FACS tubes (BD Falcon # 353054), centrifuged at 300 x g for 10 min, and washed with staining buffer. [DPBS without Ca^2+^ or Mg^2+^ (Gibco #14200–075), 1% HIFBS, 0.09% sodium azide, pH 7.2]. Cells were incubated at 4°C for 30 min with Fc Block (BD #553142) at 1μg/million cells in 100 μL of staining buffer. Fc block was washed off with staining buffer and cells pelleted at 300 x g followed by resuspension in FBS (Hyclone #SH30071.03) at 50 μL/2 million cells.

Cells were stained and fixed using the PerFix no centifuge Kit (Beckman Coulter # B10825). The cells were fixed for 15 min at RT with 2.5 μl fixative reagent R1, followed by a 30 min incubation with 150 μL of permeabilizing reagent R2 with antibodies at 1:100 dilution (PerCP-CD45, Clone 30-F11, Biolegend #103130; APC-CD11b, Clone M1/70, Biolegend #101212; APC-Cy7-Ly6G/Ly6C, Clone RB6-8C5, Biolegend #108424; PE-Cy7-Ly6G, Clone 1A8, Biolegend #127618; PE-TNF-alpha, Clone MP6-XT22, eBioscience #127321). All antibodies were titrated prior to use. After staining, cells were washed with 2 mL of fixative reagent R3, and resuspended in 400 μL of staining Buffer. Samples were read on a BD FACS Canto II and 50,000 events collected per sample. The resulting FSC files were analyzed on FlowJo V10 (Treestar, San Jose, CA) with compensation matrix set by BD Bioscience CompBeads (# 552843).

### Western Blot

Standarized protein samples were mixed with 2x loading dye, boiled and frozen at -20°C until use. Proteins (20ug) were resolved on 7.5% TGX gels (Biorad # 456–1025) and blotted onto a nitrocellulose membrane, which was blocked in 5% dry-milk and incubated overnight in 1:1000 anti-BACE1 primary antibody (Sigma, B0806). Syngene G:box using GENEsys automatic control program was used to image the blot after incubation with the ECL reagents (SuperSignal West Pico # 34080, ThermoFisher Scientific; ECL Prime RPN2232, GE Healthcare). BACE1 values were quantified using Image J (NIH) and normalized to beta-actin.

### Isoindolin-1,3 dithione (IDT) synthesis and thin layer chromatography detection of IDT in food pellets

Phthalimide (isoindoline-1,3-dione, 25.0 g, 0.17 mol) was dissolved in 400 mL of dry toluene in a 2 L round bottom flask, and Lawesson’s Reagent (137.0 g, 0.34 mol) was added in three portions, five minutes apart. The solution was then heated to reflux. During the course of the reaction the solution changes color from light yellow to orange, to green/brown and then finally to black. The reaction was monitored by thin layer chromatography (TLC; 8:2 ethyl acetate/hexane), and upon complete disappearance of starting phthalimide (approximately 4 h), the heating was stopped and the solution was allowed to cool for about 30 minutes. The reaction mixture was filtered while still warm, leaving behind a dark residue, while the filtrate contained most of the product. The residue left behind was checked by TLC for the presence of product, and if present, the residue was washed with a small amount of ethyl acetate. The solvent in the filtrate was then removed by rotary evaporation to leave a black tar-like solid (approximately100 g). This solid was then re-dissolved in approximately 500 mL of ethyl acetate. Silica gel was then added to the solution, and the solvent was removed by rotary evaporation to leave the solid adsorbed onto silica gel. This silica gel was then dry loaded onto a silica gel column for flash column chromatography purification using 95:5 Hexane/EtOAc as the mobile phase. Fractions containing the desired product were collected together, and the solvent was removed by rotary evaporation to yield 22 g of the crude product as a purple/black solid. The product was then recrystallized from ethyl acetate to obtain the pure product as purple, needle shaped crystals (19.0 g, 63% yield). Mp = 197–199°C. TLC (8:2 ethyl acetate/hexane), *R*_*f*_ = 0.8. ^1^H NMR (400 MHz, CDCl_3_): *δ* 9.73 (s, 1H), 7.88 (d, *J* = 5.7 Hz, 2H), 7.77 (d, *J* = 5.7 Hz, 2H). ^13^C NMR (100 MHz, CDCl_3_) 197.32, 135.08, 133.59, 123.13. HRMS (ESI) m/z for C_8_H_6_NS_2_ [M+H]^+^ calcd 179.9936, found 179.9939.

Three samples of feed pellets were analyzed: Sample 1: Control feed pellet lacking IDT; Sample 2: Feed pellet containing IDT immediately after removal from the freezer; and Sample 3: Feed pellet containing IDT which had been stored at room temperature for a week. The pellets were crushed and ground to a fine powder using a mortar and pestle, and equal weights (2.5 g) of each sample was taken for analysis. The powders were stirred in acetonitrile (25 mL) for 20 minutes, and filtered. The residue was re-suspended in acetonitrile (25 mL), stirred for another 20 minutes, and filtered again. The two filtrates were combined, and the solvent (acetonitrile) was removed by rotary evaporation. To the residue left in the flask, acetonitrile (5 mL) was added to re-dissolve any extracted IDT. The solutions were analyzed by TLC (80:20 hexane:ethyl acetate) and HPLC (Sonoma C18 column reverse phase column, 5μ, 100 Å. Mobile phase: 65:35 acetonitrile:water, flow rate 1 mL/min).

### BV2 Cell Culture & Lipopolysaccharide treatment

BV2 cells (gift from Todd Morgan, University of Southern California [20]) were maintained in culture medium (CM) consisting of Dulbecco’s modified Eagle medium (DMEM + L-Glutamine, ATCC Cat #30–2002) with 10% fetal bovine serum (FBS, ATCC Cat #30–2020), penicillin/streptomycin (10,000 IU-10,000 μg/mL; ATCC Cat#30–2300) in a 5% CO_2_ incubator. Plated cells (30,000 cells/well; 96 well plate) were grown in CM. In all experiments, cells were treated with the indicated concentrations of IDT or vehicle (DMSO) in the absence or presence of lipopolysaccharide (LPS; 100 ng/mL; serotype O55:B5 from E. Coli) in serum-free CM. Final concentration of DMSO was 1%. The supernatant of the BV2 cells was collected at 24 h after LPS ± IDT stimulation, briefly centrifuged to remove floating cells and debris and stored at -20°C prior to ELISA analysis. Lactate dehydrogenase (LDH) assay (Promega CytoTox96® non-radioactive cytoxicity assay) was performed in triplicate on CM as per manufacturer’s protocol.

### Statistics

All data were graphically presented as mean ± SEM unless otherwise specified. In the case of single mean comparisons, data were analyzed by two-tailed unpaired t-tests or Mann-Whitney tests appropriate to data distributions. In case of multiple comparisons, data were analyzed by one-or two-way ANOVA with *post-hoc* Bonferroni or Dunnett’s multiple comparisons tests using GraphPad Prism Software (GraphPad version 5).

## Results

### IDT possesses potent anti-TNFα activity *in vitro*

**Fig 1:**
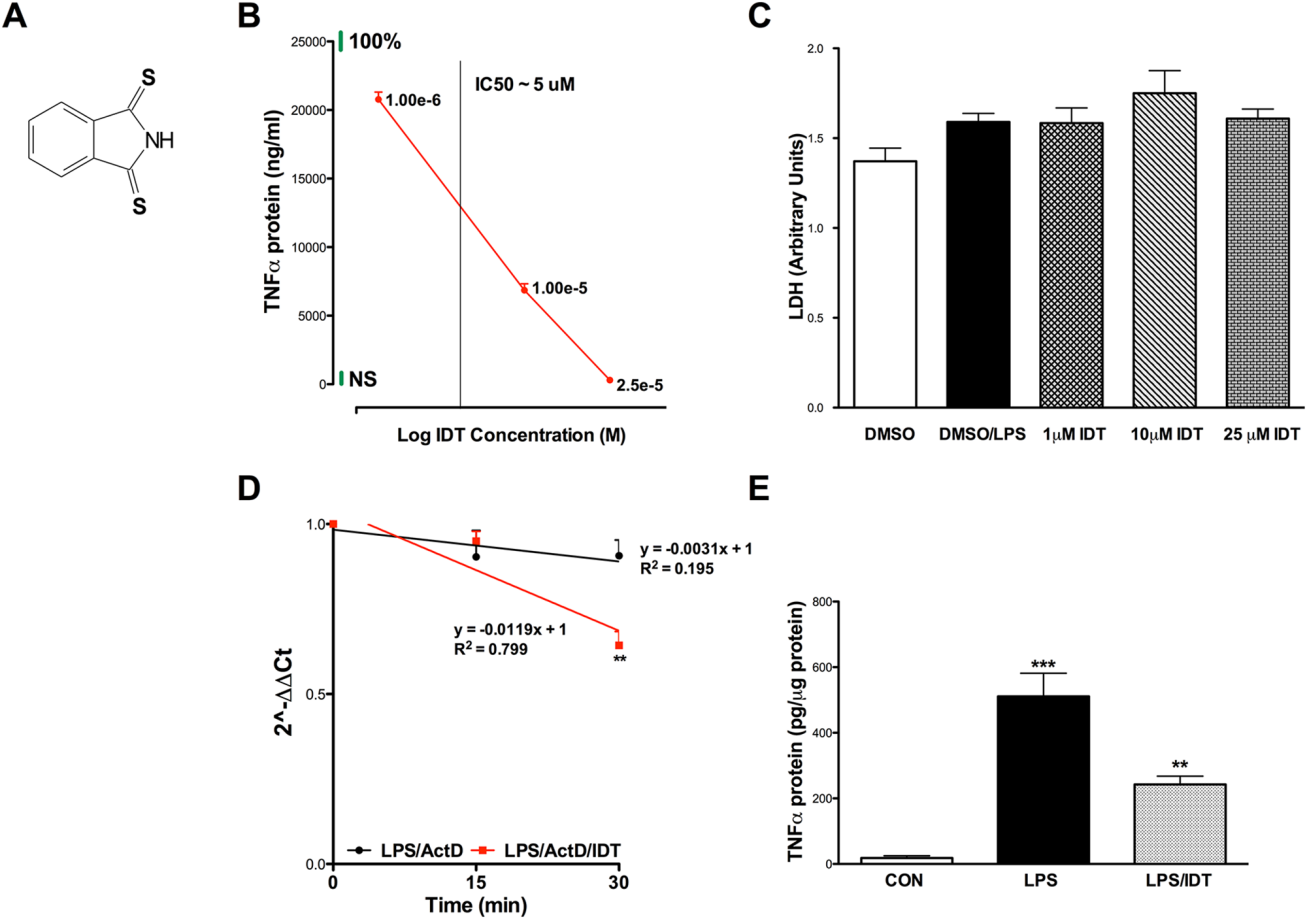
Effects of isoindolin-1,3 dithione (IDT) on TNFα protein and mRNA. (A) Structure of IDT. (B) Initial studies in BV2 cells demonstrate that IDT is effective at attenuating LPS-induced TNFα release into culture media (One-way ANOVA, p < 0.0001. p < 0.001 LPS vs. all IDT doses) with an IC_50_ value of ~5 μM. Green bars at the top of the y-axis and right side of the x-axis indicate the 100% LPS and no LPS (NS) responses, respectively. n = 4/group. (C) Lactate dehydrogenase (LDH) release into the culture media was measured after 24 hr exposure to LPS ± IDT. No significant increase in LDH was detected compared to DMSO control. (D) Effect of IDT on TNFα mRNA stability in BV-2 cells. Cells were pre-treated for 4 hr with 100 ng/mL LPS followed by treatment with 25 μM IDT and 5 mg/mL actinomycin D (ActD) and collected at intervals of 15 min. Total RNAs were isolated and TNFα mRNA was quantified using qPCR. Values represent mean ± s.e.m. for two separate experiments performed in triplicate and relative quantification was performed using the Rq method 2 ^-ΔΔCt^. Asterisks indicate significant difference from LPS/ActD-treated BV-2 cells (**P < 0.01). n = 3/group. (E) IDT inhibited LPS-stimulated Cortical TNFα protein expression in vivo. Mice were treated by oral gavage with a single dose (50 mg/kg) of IDT 30 minutes prior to a peripheral 5 mg/kg dose of LPS (i.p.). Cortical tissue was harvested 4 hours after LPS injection. n = 6/group. One-way ANOVA, p < 0.001; ***p < 0.001 vs. Con and **p < 0.01 vs. LPS. Data represent mean ± SEM of n = 3/group.

The immortalized microglial cell line, BV2, was initially used as a screen to assess the TNFα modulating activity of IDT. BV2 cells respond similarly to primary microglial cells when stimulated with LPS and are a valid in vitro model [21]. BV2 cells exposed to LPS produced a robust TNFα response that was efficiently attenuated by increasing doses of IDT (Structure of IDT is shown in Fig 1A). Doses ranging from 1–25 μM significantly inhibited LPS induced TNFα with an IC_50_ of approximately 5 μM (Fig 1B). These results are similar to a previously published IC_50_ of 3 μM when IDT was evaluated in freshly prepared peripheral blood mononuclear cell (PBMC) cultures exposed to a 100 ng/mL, 16 hour LPS challenge [22]. Although the LPS challenge slightly increased LDH release from the BV2 cells, there was no significant toxicity evident from the addition of any IDT dose as evaluated by LDH release at the 24 hr exposure time point (Fig 1C).

Previous work evaluating a series of thiothalidomide analogues suggested that the mechanism of action for the modulation of TNFα by these compounds involved decreased TNFα mRNA stability via binding of the 3’-untranslated region (UTR) [22]. A reduction in TNFα protein suggests a potential for regulation of TNFα mRNA at the transcriptional level or by an effect on mRNA stability. We tested the latter hypothesis, assessing TNFα mRNA stability following a 4 hr LPS stimulus and in the presence or absence of IDT using actinomycin D (ActD), a well-known inhibitor of transcription. In the absence of IDT, TNFα mRNA decreased by ~7% in 30 min in the presence of ActD, whereas the association with IDT accelerated mRNA reduction to ~35% in 30 min (Fig 1D), indicating the ability of IDT to interfere with one or more processes regulating mRNA stability.

### IDT possesses potent anti-TNFα activity *in vivo*

**Fig 2:**
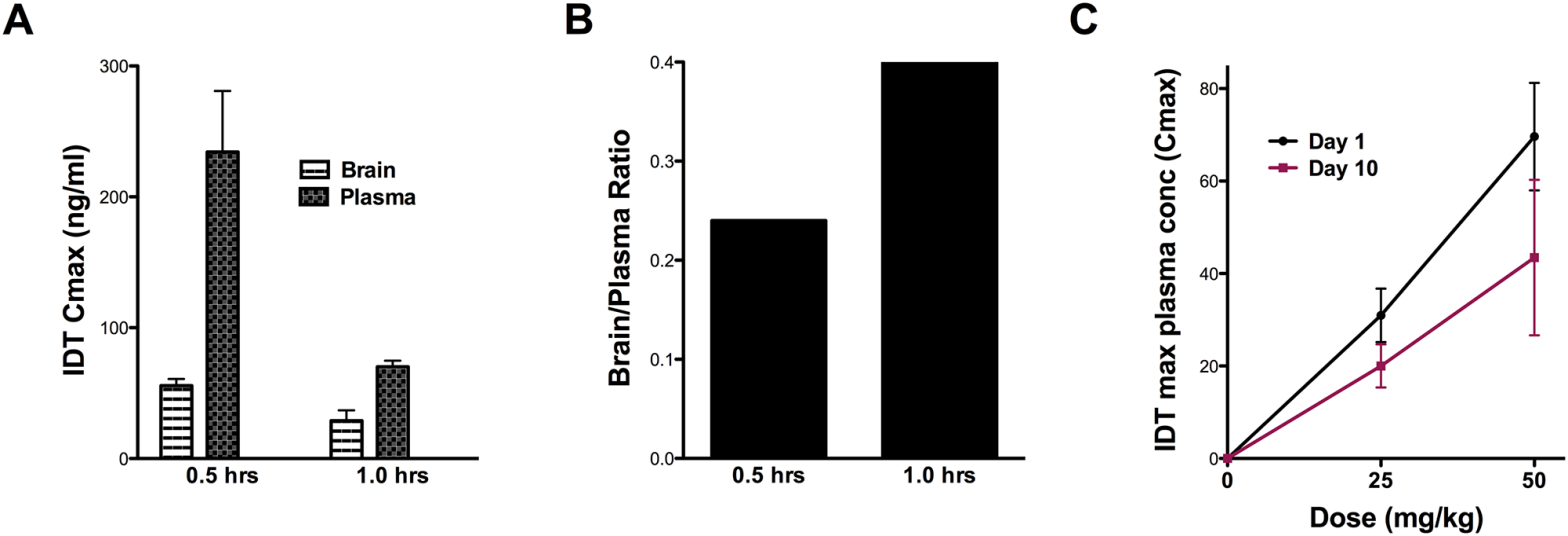
IDT pharmacokinetics. (A) Single dose of IDT (25 mg/kg; i.p.) to demonstrate plasma and brain partitioning. (B) Calculated brain to plasma ratios. (C) Plasma IDT (ng/mL) 30 min after oral delivery of doses up to 50 mg/kg (A).

We next sought to determine if IDT was an effective TNFα modulator in vivo. Expanding on our in vitro LPS studies, we gavaged C57Bl mice with a 50 mg/kg dose of IDT 30 minutes prior to a 5 mg/kg (i.p.) LPS challenge. Following a 4 hr survival period, cortical tissue was evaluated for TNFα protein levels using an Optimiser microfluidics-based immunoassay. LPS produced a robust increase in cortical TNFα protein levels and this increase was significantly reduced to 40% of LPS levels by IDT (Fig 1E), confirming that IDT is orally bioavailable and efficacious. Oral pharmacokinetic studies also confirm that detectable levels of IDT are found in plasma (Fig 2a).

Measuring the brain/plasma ratio of a drug following intraperitoneal injection or oral gavage is a common approach in CNS drug discovery projects and provides a simple ratio of drug concentration in brain and plasma. The ratio can be used to calculate a permeability coefficient, essentially as described by Ohno and colleagues [23]. It provides a simple measure of partitioning but does not take into account the presence of drug in the brain vasculature, and results are expressed as a brain/plasma ratio. Generally speaking, drugs with a permeability coefficient of >1 are considered to have good CNS distribution. We have confirmed the presence of IDT in plasma following both i.p. injection (Fig 2A) and oral gavage (Fig 2C). Further, Fig 2B demonstrates that IDT was also detected in brain and these values gave calculated brain/plasma coefficients of 0.25 and 0.4 respectively. Although these coefficient values would not qualify IDT as having good CNS distribution, this aspect may not be of high importance if modulation of the peripheral innate immune system is primarily responsible for IDT’s effects. Thus, blood concentrations of IDT may be more pertinent.

### IDT is orally bioavailable and well-tolerated

We utilized the oral route of IDT administration for studies in the 3xTgAD mouse expressing transgenic mutant human amyloid precursor protein (APP) with Swedish mutation (APPswe), transgenic mutant TauP301L and knock-in mutant presenilin-1 (PS1M146V) [13]. Because studies in 3xTgAD mice necessarily require many months of chronic dosing, we formulated IDT into the diet at 80, 200 and 400 mg/kg to achieve final daily doses of 10, 25 and 50 mg/kg based on mouse weight. The stability of IDT in feed, up to one week at room temperature, was verified by TLC/HPLC (S1 Fig). Mice were fed daily in their cage and, with few exceptions, consumed their entire dose of diet over a 24 hr period. Any remaining food was discarded and replaced daily with fresh diet.

Low dose IDT (Low; 10 mg/kg) did not alter weight over the 38 weeks of dosing in either gender (S2 Fig). Medium (Med; 25 mg/kg) and High doses (50 mg/kg) did alter weight over the dosing period (6% and 25% reduction in weight for Med and High doses respectively in males and a 15% reduction for both Med and High doses in females at the 38 week dosing end-point). However, despite reduced weight increases over time in some groups, mice appeared healthy, active and were capable of performing cognitive tasks.

### IDT dose-dependently improves cognitive performance in the 3xTgAD mouse

**Fig 3:**
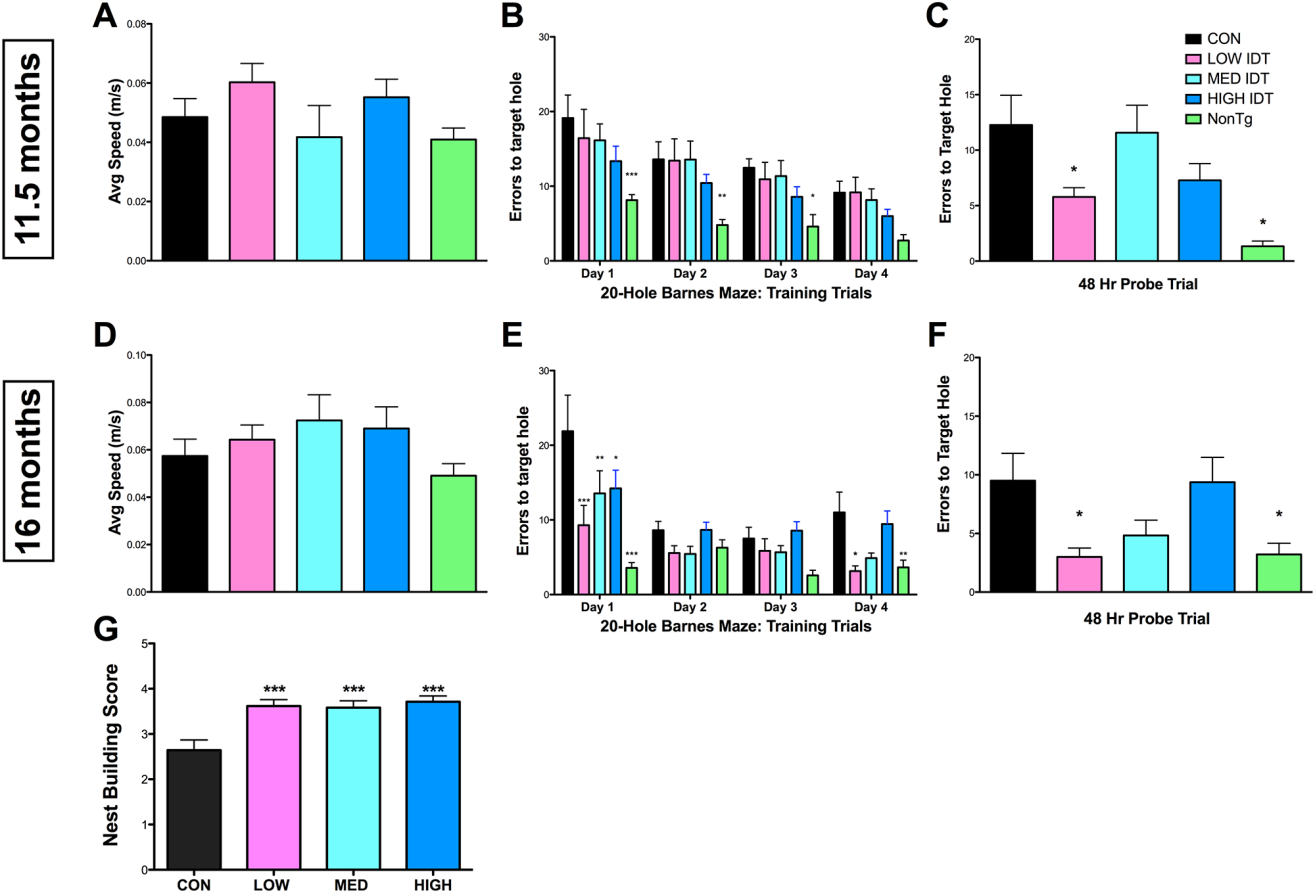
Behavioral analysis. Barnes maze testing was used to longitudinally examine the effects of IDT on learning and memory at 11.5 months (A-C) and 16 months (D-F) of age. Four days of training was followed 48 hrs later by a retention probe trial. There were no significant differences in average speed at either time point examined (A/D). At 11.5 months of age, all treatment groups were able to learn the location of the escape hole (B). Repeated measures two-way ANOVA of the acquisition phase revealed a significant effect of time (F_3, 195_ = 17.06, P < 0.001) and treatment (F_4, 195_ = 7.8, P < 0.001). NonTg mice performed significantly better than Con 3xTgAD mice (*p< 0.05, **p<0.01, ***p<0.001). One-way ANOVA of the 48 hr retention probe trial (C) revealed a significant treatment effect on memory retention (F_4, 71_ = 6.184, P < 0.0001). Low IDT treatment significantly improved spatial memory retention compared to Con 3xTgAD mice (*p < 0.05). NonTg performance on the probe trial was also significantly improved from Con 3xTgAD mice (***p < 0.001). Data represent mean ± SEM of n = 14-15/group. At 16 months of age, all treatment groups were again able to learn the location of the escape hole. Repeated measure two-way ANOVA of the acquisition phase (E) revealed a significant effect of time (F_3, 126_ = 17.06, P < 0.001) and treatment (F_4, 126_ = 11.65, P < 0.001). The interaction of Time x Treatment was significant (F_12, 126_ = 3.18, P = 0.005). NonTg and IDT treated mice had improved performance compared to Con 3xTgAD mice (*p< 0.05, **p<0.01, ***p<0.001). One-way ANOVA of the 48 hr retention probe trial (F) revealed a significant treatment effect on memory retention (F_4, 43_ = 4.538, P = 0.0042). NonTg and Low IDT mice performed significantly better on the spatial memory retention trial compared to Con 3xTgAD mice (*p < 0.05) with Med IDT mice also showing improved performance. Data represent mean ± SEM of n = 6-14/group. (G) Nesting behavior. Mice were given nesting materials and 24 hrs to build a nest. Nests were scored on a scale from 0–5. All IDT doses improved scores vs. Con 3xTgAD mice (***p < 0.001). Data represent mean ± SEM of n = 12–14/group.

Cognitive performance was evaluated by Barnes maze at two time points after initiation of IDT dosing; 11.5 months of age (~20 weeks of IDT dosing) and 16 months of age (~38 weeks of IDT dosing). Fig 3 shows the Barnes maze data from these two time points. An additional age-matched, wildtype group (NonTg; background strain of the 3xTgAD mice) was included for reference. Graphs A and D show the average speed of the mice as they navigated the Barnes maze during the acquisition/learning phase of the task and demonstrates a lack of effect of either transgene or IDT on average speed at either time point. At 11.5 months of age, all 3xTgAD mouse groups were able to learn the location of the escape hole over the four-day training period with no significant differences in errors between treatment groups. NonTg mice learned the location of the escape hole sooner than the 3xTgAD groups and had overall fewer errors. 48 hrs following the acquisition phase, a probe trial was conducted to assess memory for the escape hole location. Only the Low diet group and NonTg group had significantly fewer errors compared to Con diet 3xTgAD mice. At 16 months of age, although all treatment groups were ultimately able to learn the location of the escape hole over the four-day training period, the Low IDT group performed significantly better than the Con, Med and High diet groups but similarly to the NonTg group. The 48 hr probe trial again revealed a significant reduction in errors in the Low diet group, performing comparably to the NonTg group. There was a trend to reduction in probe trial errors in the Med diet group but this did not reach significance.

Previous studies have demonstrated that hippocampal lesioned mice used less nesting material and built poorer nests [24]. This result is consistent with other studies examining hippocampal damage in gerbils and rats [25–28]. Nest building competency scores in the IDT-treated mice were significantly greater than those of the Con diet group (Fig 3G). These data are another indicator that long-term IDT dosing does not compromise the overall health of the mice.

### IDT dose-dependently increases infiltrating neutrophils and reduces TNFα protein in the CNS

**Fig 4:**
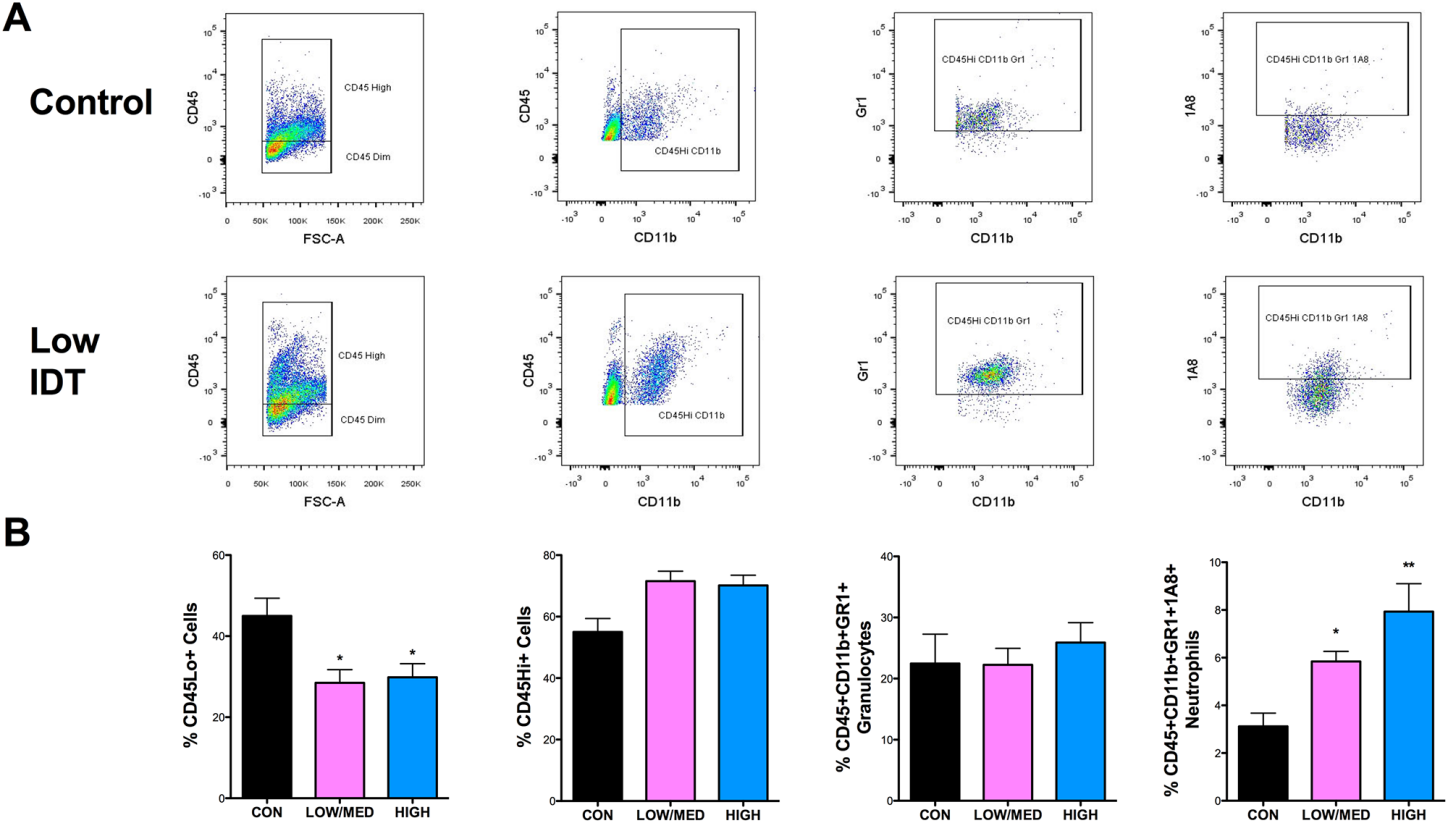
Flow cytometric analysis of leukocytes recovered from the brains of Con and IDT-treated 3xTgAD mice. (A) Representative flow cytometry profiles of the cell surface markers and gating strategy are shown for Con and Low IDT brains. (B) The percentage of leukocytes (CD45Hi+), microglia (CD45Dim+), granulocytes (CD45Hi+/CD11b+/GR1+) and neutrophils (CD45Hi+/CD11b+/GR1+/1A8+) among all cells analyzed across the four treatment groups. One-way ANOVA of the neutrophil populations revealed a significant IDT effect (F_2, 10_ = 6.468, P = 0.0023). Both Low/Med and High IDT treatment groups significantly increased neutrophil infiltration into the brain relative to Con 3xTgAD brains (*p < 0.05; **p < 0.01).

**Fig 5:**
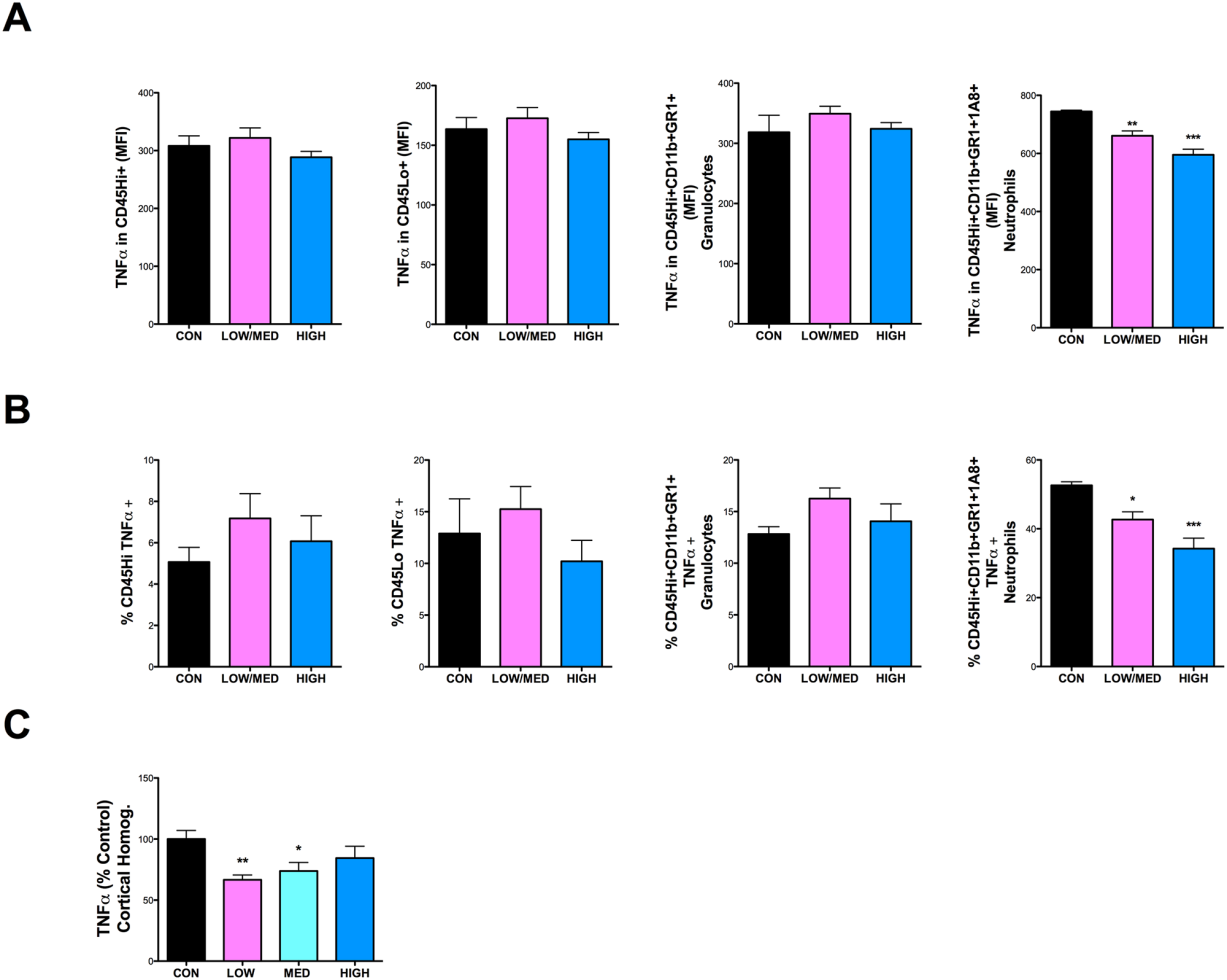
TNFα in leukocyte/microglial populations and in whole cortex. (A) Mean fluorescence intensity (MFI) of TNFα in leukocytes (CD45Hi+), microglia (CD45Dim+), granulocytes (CD45Hi+/CD11b+/GR1+) and neutrophils (CD45Hi+/CD11b+/GR1+/1A8+). TNFα intensity was significantly lower in neutrophils both in the Low/Med (**p < 0.01) and High IDT (***p < 0.001) dose groups compared to Con 3xTgAD group. (B) The percentage of cells showing a positive TNFα signal was also analyzed in the same cell groups. There was an ~13% (Low/Med IDT; 8p < 0.05) and ~18% (High IDT; ***p < 0.001) reduction in the cells positive for TNFα versus Con 3xTgAD group. (C) Whole cortical homogenates were also analyzed for TNFα levels. There was a ~40% (Low IDT; **p < 0.01) and ~30% (Med IDT; *p < 0.05) reduction in total cortical TNFα versus Con 3xTgAD group.

Flow cytometry was used to quantify and phenotype blood-derived leukocytes and CNS-resident microglia in 3xTgAD mice. As there was no statistical difference between Low diet and Med diet flow cytometry data, those two groups were combined for analysis. The gating strategy used to evaluate various cell populations is shown in Fig 4A. A representative Con diet and Low IDT mouse are shown. IDT treatment at any dose did not significantly reduce the % of CD45Hi+(infiltrating leukocytes) or CD45Hi+/Cd11b+/GR1+ (granulocytes) but did increase CD45Hi+/Cd11b+/GR1+/1A8+ (neutrophils) and reduce (~12%) the CD45Dim+ (microglia) in both the Low/Med and High diet doses (Fig 4B). Fig 5 shows the data for TNFα expression in the same populations. Fig 5 row A shows the mean fluorescence intensity (MFI) for TNFα. Only the neutrophil population showed a decrease in TNFα MFI with increasing IDT dose. Fig 5 row B shows the percentage of the isolated populations that were TNFα+. The total number of neutrophils that were TNFα+ decreased with increasing IDT dose. Fig 5, graph C shows TNFα protein levels in whole cortex homogenates where the Low dose IDT was most effective, perhaps reflecting a reduction in neuronal and extracellular space levels of TNFα also with Low and Med dose IDT.

**Fig 6:**
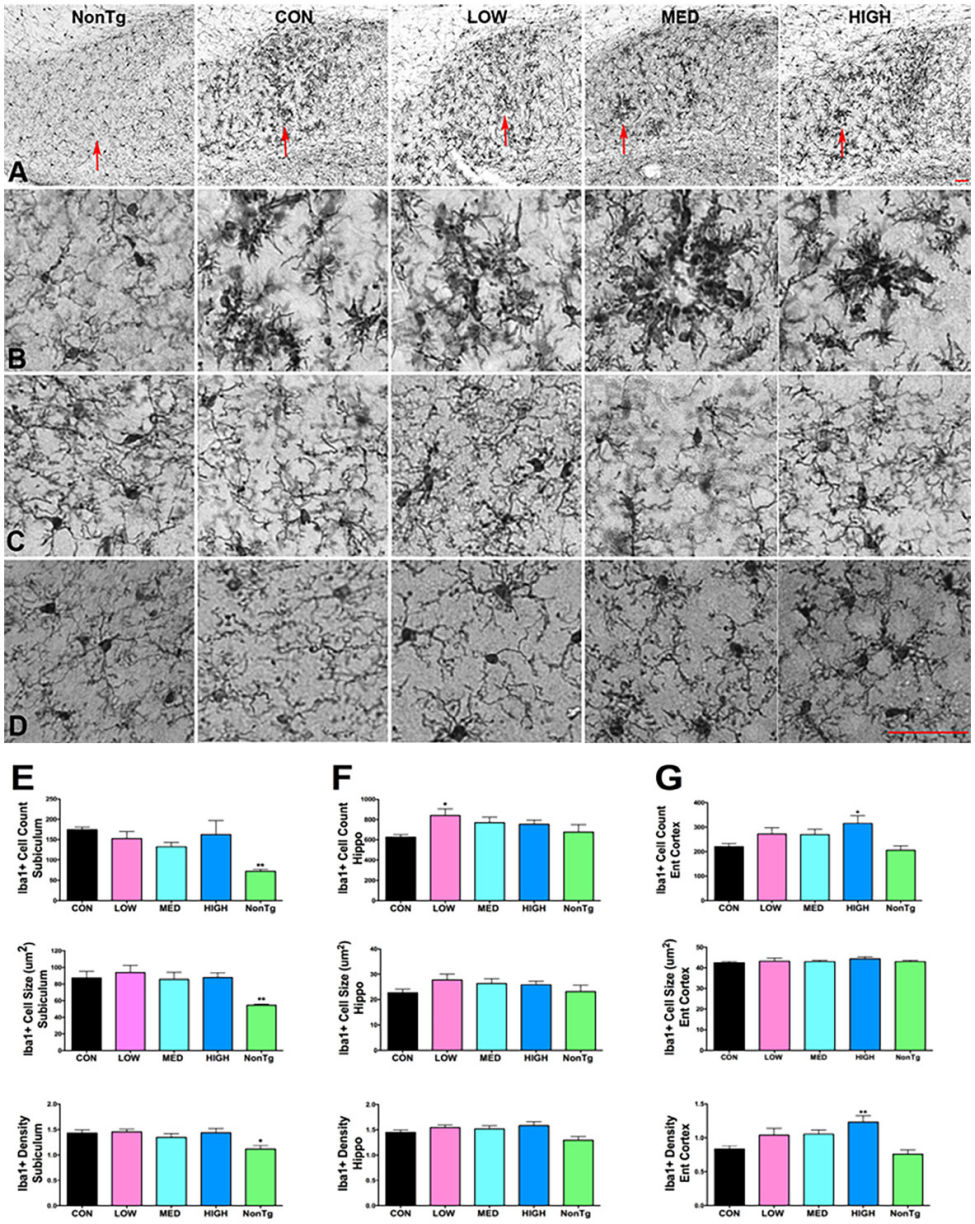
Iba-1 staining. 50 μm sections, every 12^th^ section through the length of hippocampus, were stained and analyzed (total of 5 sections/mouse). Pictures show representative sections from each treatment group for three different brain regions: Subiculum, hippocampus and entorhinal cortex. (A) 10x magnification of the subiculum. Red arrows point to regions shown in (B; 20x magnification). 20x magnification of hippocampus (C; CA1 region) and entorhinal cortex (D). Sections were analyzed for cell counts, cell size (μm^2^) and staining density for each region (E-F). *p < 0.05 vs. Con. Data represent mean ± SEM of n = 6–10/group. Bar equals 50 μm.

In addition to isolation of CNS microglia and leukocytes by flow cytometry, we examined microglia/macrophages in three regions (hippocampus, subiculum and entorhinal cortex) histologically by Iba1 immunocytochemistry (ICC) (Fig 6). The subiculum is a region of high AD pathology load in the 3xTgAD mouse and displayed the most significant differences in Iba1+ cell count, cell size and density compared to NonTg mice (Fig 6E). Iba1 ICC revealed overall stable numbers of Iba1+ cells across all brain regions at all IDT doses with the exception of increased numbers of Iba1+ cells in the hippocampus with low dose IDT and in the entorhinal cortex with high dose IDT (Fig 6F and 6G). IDT at any dose did not significantly change the density of Iba1 staining or the Iba1+ cell size (Fig 6E–6G) with the exception of high dose IDT in the entorhinal cortex. Consistent with the flow cytometry data, IDT treatment did not dramatically alter the total Iba1+ cell population in the brain. Although there were no dramatic changes in Iba1+ cell numbers following IDT treatment, it is interesting to observe that IDT altered the spatial orientation of Iba1+ cells in the subiculum (see red arrows, Fig 6A). In the Con 3xTgAD mice, cells appear randomly distributed. With IDT, Iba1+ cells appear to coalesce into circular structures that grow more compact with increasing IDT dose.

The impact of IDT on peripheral blood cytokines and spleen cell populations and TNFα was also evaluated. S3 Fig shows the spleen flow cytometry data using the same cell population markers as in the brain flow cytometry data. There were no significant changes in: 1) spleen cell populations (S3 Fig, panel A), 2) TNFα levels in the spleen cell populations (S3 Fig, panel B) or 3) % of cells that were TNFα+ in those cell populations (S3 Fig, panel C). We also evaluated a four-panel of cytokines (TNFα, IL-6, MCP-1 and CXCL2) to determine any IDT-mediated changes in these serum cytokines (S4 Fig). There was a trend towards decreasing TNFα and IL-6 with Low dose IDT but no effect of higher IDT doses or any effect on MCP-1 and CXCL2.

### Modulation of neuroinflammatory phenotype in innate immune cells

**Fig 7:**
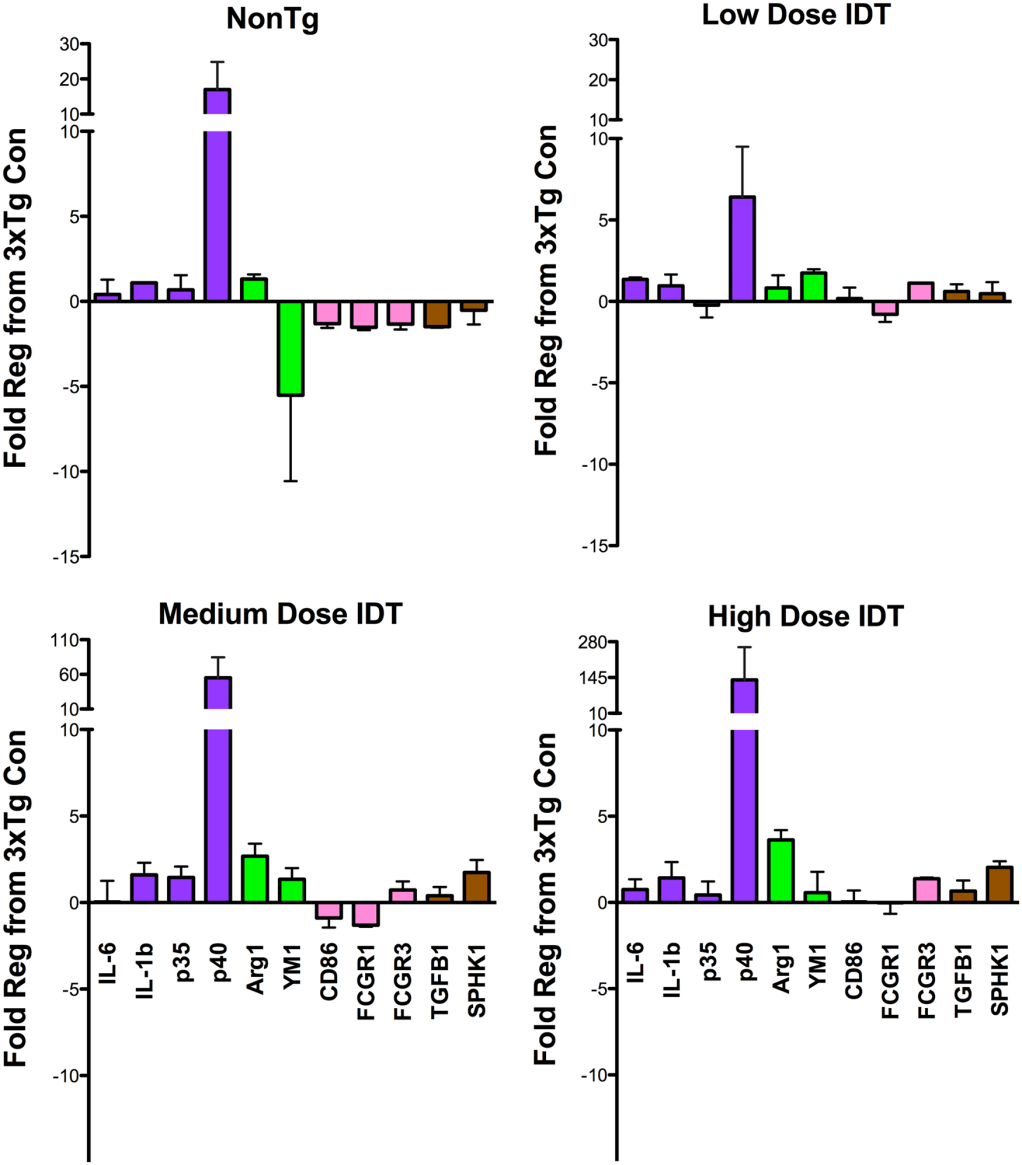
IDT increases p40 gene expression. Relative gene expression for a diverse array of phenotypic markers. Data are shown as fold regulation relative to Con 3xTgAD mice. ARG1, arginase 1; FcγR, Fc gamma receptor; IL, interleukin; SPHK1, sphingosine kinase 1; TGF, transforming growth factor. All gene fold regulation values are shown in Table 1 as mean ± SEM of N = 3–6/group.

To determine changes in innate cell phenotype, cortical RNA was isolated and real time PCR performed for several genes specific to different markers of macrophage phenotype. Data are shown as fold regulation compared to 3xTgAD Con diet mice (Fig 7). Mean ± SEM and p values for all data in Fig 7 are shown in Table 1. The top left graph compares NonTg mice to 3xTgAD Con mice. The most obvious difference is that NonTg mice have significantly more p40 (IL-12b) which is upregulated more than 15 fold compared to 3xTgAD Con mice. IDT caused a dose-dependent rise in p40 levels with the NonTg p40 level falling between the Low and Med IDT expression levels. With the exception of p40, fold changes for other markers were in the ± 1 to 5 fold range).

**Table 1:** Fold regulation (compared to 3xTg Con mice) values for real time PCR.

Gene	LOW IDT	p value	MED IDT	p value	HIGH IDT	p value	NonTg	p value
**IL-6**	1.36 ± 0.12	0.19	0.04 ± 1.22	0.47	0.75 ± 0.59	0.2	0.40 ± 0.88	0.86
**IL-1β**	0.96 ± 0.69	0.29	1.6 ± 0.70	0.25	1.42 ± 0.93	0.34	1.09 ± 0.016	**0.05**
**IL-12a**	-0.23 ± 0.75	1.00	1.45 ± 0.64	0.17	0.43 ± 0.78	0.49	0.68 ± 0.85	0.22
**IL-12b**	6.4 ± 3.1	0.14	55.23 ± 29.29	**0.04**	135.8 ± 123.4	**0.03**	16.99 ± 7.85	**0.05**
**ARG1**	0.82 ± 0.78	0.19	2.68 ± 0.72	**0.009**	3.63 ± 0.57	**0.03**	1.31 ± 0.27	0.63
**YM1**	1.75 ± 0.23	**0.03**	1.35 ± 0.64	0.13	0.57 ± 1.20	0.89	-5.52 ± 5.05	0.15
**CD86**	0.18 ± 0.67	0.73	-0.89 ± 0.55	0.35	0.03 ± 0.67	0.89	-1.31 ± 0.25	0.40
**FcγR1**	-0.79 ±0.47	0.11	-1.31 ± 0.09	**0.04**	-0.024 ± 0.64	0.89	-1.52 ± 0.17	**0.05**
**FcγR3**	1.12 ± 0.03	0.28	0.72 ± 0.50	0.76	1.38 ± 0.066	0.49	-1.34 ± 0.31	0.40
**TGFβ1**	0.61 ± 0.45	0.73	0.39 ± 0.50	0.76	0.66 ± 0.61	0.34	-1.49 ± 0.06	**0.05**
**SPHK1**	0.48 ± 0.70	0.41	1.74 ± 0.73	0.11	2.04 ± 0.35	0.11	-0.52 ± 0.84	0.40

Data shown as mean ± SEM. Bold font indicates P < 0.05 compared to CON diet 3xTgAD mice. ARG1, arginase 1; FcγR, Fc gamma receptor; IL, interleukin; SPHK1, sphingosine kinase 1; TGF, transforming growth factor. N = 3–6/group.

### IDT reduces Thioflavin-S staining, insoluble amyloid levels and PHF tau

**Fig 8:**
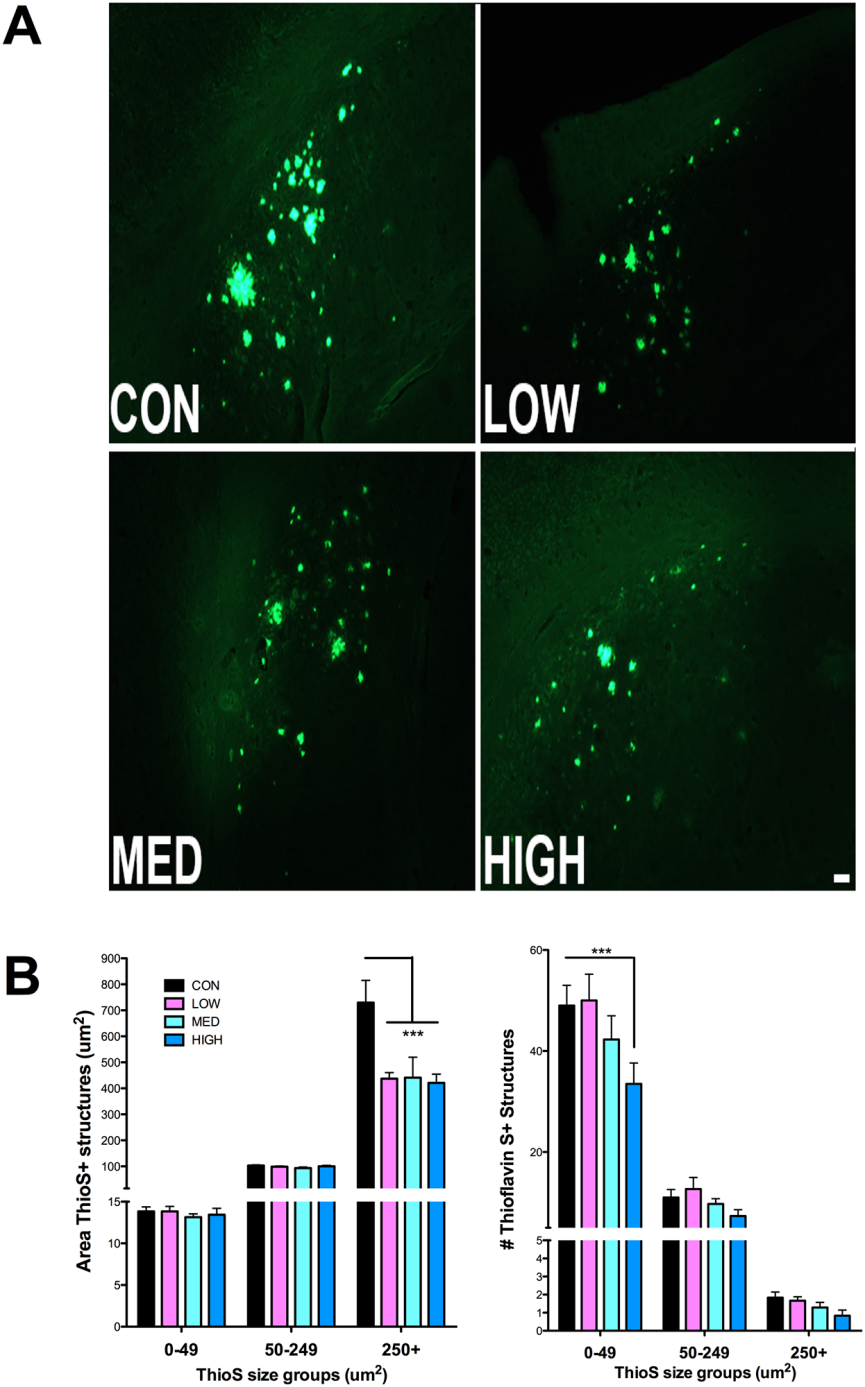
Thioflavin-S fluorescence. (A) Representative Thioflavin-S plaques (10x magnification; Bar = 50 μm). (B) Thioflavin-S positive plaques in Control diet and IDT fed 3xTgAD mice were quantified as area (μm^2^) and counts. Three different categories of ThioS+ structures were quantified according to size ranges of 0–49 μm^2^, 50–249 μm^2^ and 250+ μm^2^. Two-way ANOVA of the area measurements revealed a significant effect of ThioS category (F_2, 44_ = 223.6, P < 0.0001) and treatment (F_3, 44_ = 5.588, P = 0.0052). The interaction of category x Treatment was significant (F_6, 44_ = 5.54, P = 0.0002). Two-way ANOVA of the ThioS+ counts revealed a significant effect of ThioS category (F2, 44 = 367.3, P < 0.0001) but not treatment (F_3, 44_ = 2.693, P = 0.072). The interaction of category x Treatment was significant (F_6, 44_ = 2.518, P = 0.0358). * p < 0.001 versus Con. Data represent mean ± SEM of n = 6–7/group.

The Thioflavin-S method detects dense plaques containing proteins in the ß-sheet conformation. These plaques are numerous and often very large in the subicular region of aging 3xTgAD mice. Thioflavin S+ structures were analyzed by size groups of 0–49, 50–249 and 250+ μm^2^. IDT effects were specific to the large (>250 μm^2^) structures with a statistically significant reduction in area of these large ThioS+ structures with all doses of IDT in the subiculum of 3xTgAD mice (Fig 8B left). High dose IDT also reduced the numbers of small (0–49 μm^2^) ThioS+structures (Fig 8B right).

**Fig 9:**
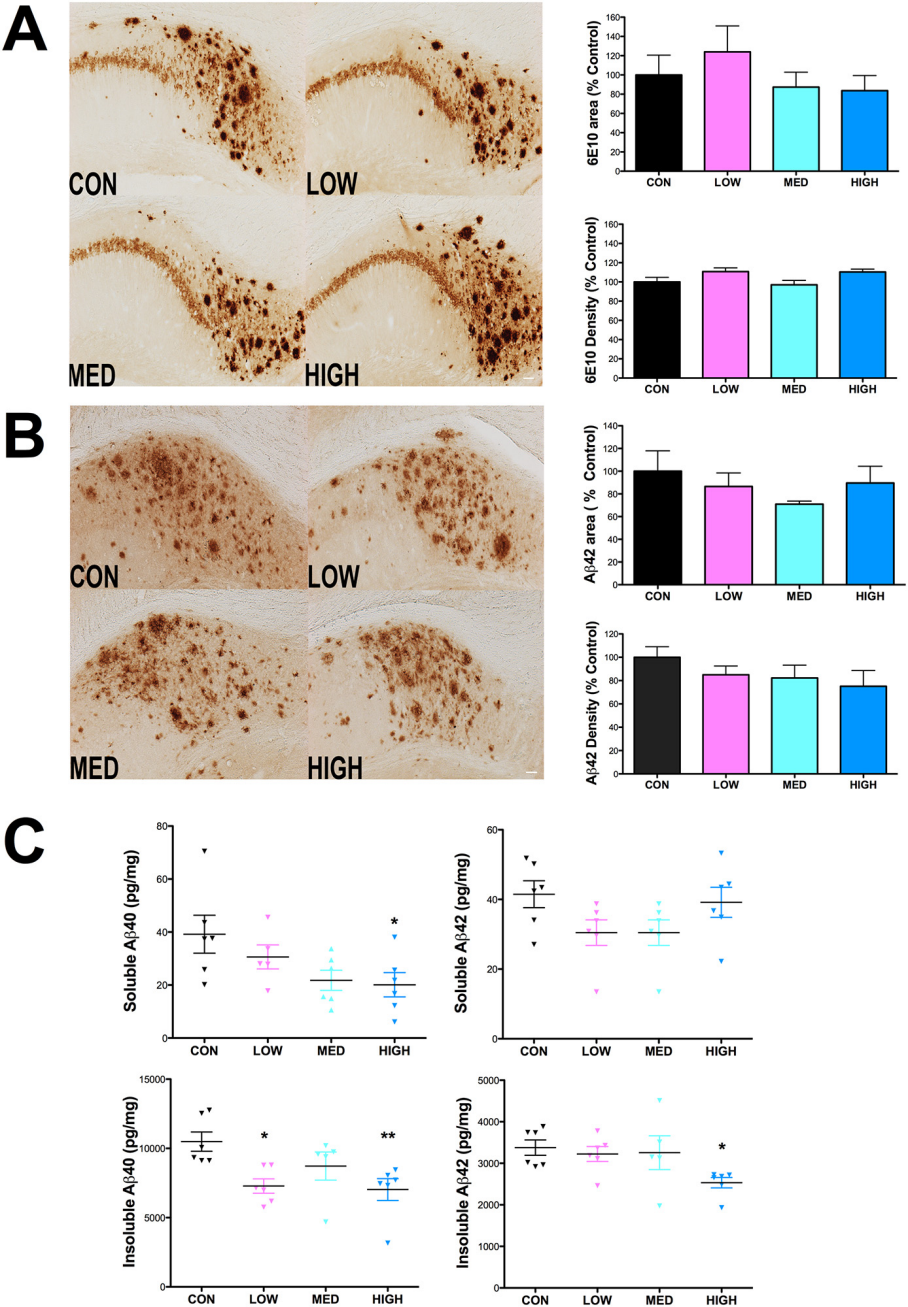
Effect of IDT effect on Aβ. Following 38 weeks of IDT treatment, analysis of 6E10 (APP/Aβ) or Aβ42 immunolabeling in the hippocampus of 16 month-old 3xTgAD mice was performed on 50 μm sections, every 12th section through the hippocampus, by densitometry and % area covered by immunostaining. (A) Representative pictures of 6E10 staining in each treatment group (10x magnification; Bar equals 50 μm) with corresponding % Area and densitometry. Similarly, Aβ42 immunolabeling and quantification is shown in (B). Elimination of the primary antibody from the staining protocol resulted in loss of specific staining (data not shown). Data represent mean ± SEM of n = 8–10/group. (C) Results from soluble and insoluble hippocampal fractions analyzed by Aβ40 or 42-specific ELISA. One-way ANOVA did not reveal an overall significant effect of IDT treatment on either soluble Aβ40 (p = 0.0604) or Aβ42 (p = 0.1181). High dose IDT did significantly reduce soluble Aβ40 versus Con (*p < 0.05). There was an overall significant effect of IDT dose on insoluble Aβ40 levels (p = 0.0135) but insoluble Aβ42 did not quite reach significance (p = 0.0649). Both Low and High dose IDT reduced insoluble Aβ40 versus Con (*p< 0.05 and **p< 0.01). High dose IDT reduced insoluble Aβ42 (*p<0.05).

Consistent with prior reports [29], immunohistochemistry utilizing the 6E10 monoclonal anti-APP/Aβ antibody [29], 6E10 revealed both plaque-like and intracellular immunoreactivity in the 3xTgAD mouse brain that was visible both in neuronal parykarya and axons (Fig 9A). There was not a significant reduction in the % area covered by 6E10+ structures in the hippocampus or in 6E10 labeling intensity in mice that had received any dose of IDT (Fig 9A). Although there was a trend towards reduction in Aβ42 labeling in the hippocampus following IDT treatment, this did not reach significance for either % area or density of Aβ42 labeling (Fig 9B).

ELISA analysis was performed on soluble and insoluble hippocampal fractions (Fig 9C). There was a trend towards reduction in soluble Aβ40 and 42 and with all IDT doses with the high IDT dose reaching significance for soluble Aβ40. The most significant effect was a reduction in insoluble Aβ40 with both low and high IDT doses. There were small reductions in insoluble Aβ42 with the high IDT dose reaching significance.

**Fig 10:**
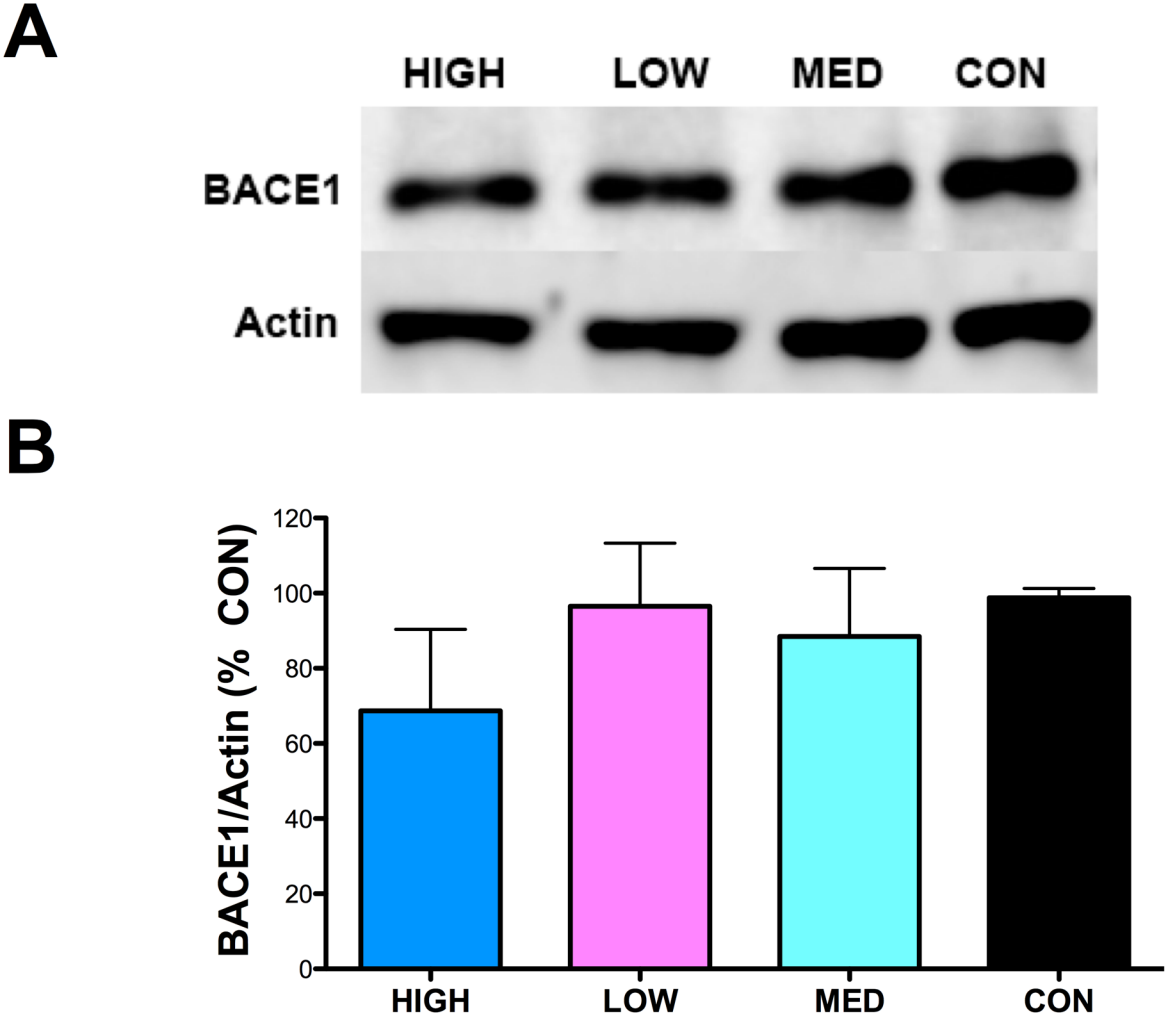
BACE1 Protein. (A) Representative western blot of hippocampal homogenates for BACE1. (B) Densitometry results for BACE1 and Actin were obtained and the changes in BACE1/Actin expressed as % Control. No significant effect of IDT was observed.

β-Secretase-1 (BACE1), the β-site amyloid precursor protein (APP) cleaving enzyme, is an aspartic protease that promotes Aβ generation [30] and TNFα signaling pathways have been implicated in the regulation of BACE1 activity [31–34]. BACE1 proteins levels were determined by western blot. No significant reduction in BACE1 protein levels with any IDT dose were found (Fig 10).

**Fig 11:**
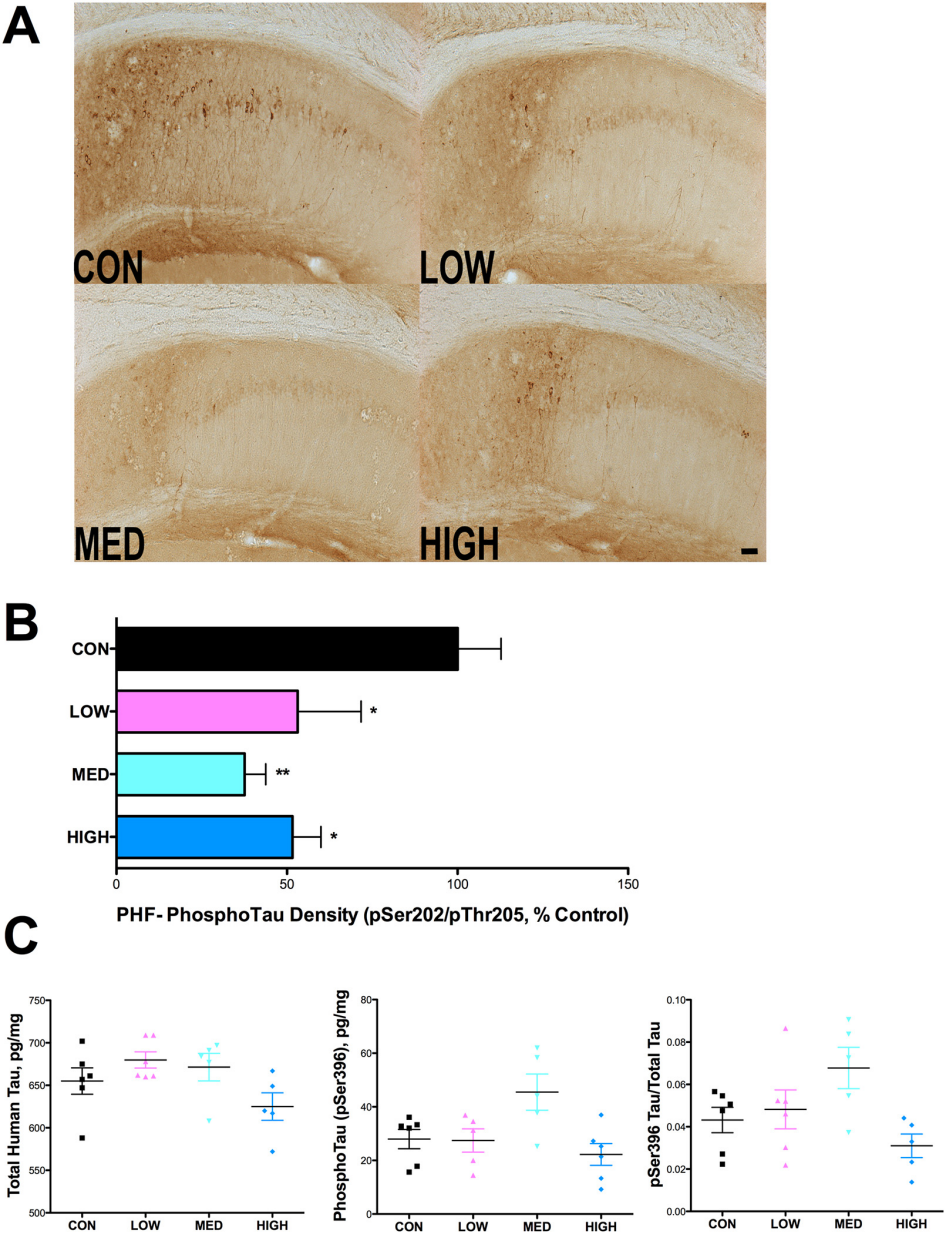
IDT reduces PHF Tau immunoreactivity in 3xTgAD mice. Following 38 weeks of IDT treatment, AT8 (pSer202/pThr205) immunolabeling in the hippocampus of 16-month-old 3xTgAD mice was performed on 50 μm sections, every 12^th^ section through the hippocampus. (A) Representative pictures of AT8 staining in each treatment group (10x magnification; Bar = 50 μm). (B) Densitometry, expressed as percentage of control, in the hippocampus was used to quantify AT8 immuno-labeling. One-way ANOVA of AT8 density data revealed a significant IDT effect (F_3, 32_ = 4.84, P = 0.0075). All three IDT doses were significantly different than Con 3xTgAD mice (*p < 0.05; **p < 0.01). Elimination of the primary antibody from the staining protocol resulted in loss of staining (data not show). Data represent mean ± SEM of n = 7–9/group. (C) Total and pSer396 tau immunoreactivity was quantified by ELISA in hippocampal homogenates. No significant differences were found in total tau or pSer396 tau. n = 6/group.

Immunohistological analysis of paired helical filament Tau was first undertaken using the well-known monoclonal antibody, AT8, which detects the phosphorylated Tau epitope on Ser202 and Thr205 [35,36] (Fig 11A). IDT treatment at all doses significantly reduced the AT8 labeling intensity in the subicular region by approximately 50–60% (Fig 11B). ELISA analysis for total human tau and phospho epitope S396 tau was performed on hippocampal samples (Fig 11C). No significant differences in total tau or pS396 tau were found.

## Discussion

The brain is a highly immunologically active organ with a dynamic innate immune system. Brain resident macrophages, microglia, play a prominent role in innate surveillance of the CNS but are not the only cellular attendants. The brain interacts with blood leukocytes, including macrophages and neutrophils, and these cells move into and out of the brain. The purpose of the innate immune system is to constantly interrogate the brain’s microenvironment and provide protection to the host from pathogens through cellular interactions and the production of cytokines. The complex nature of the inflammatory response in neurodegenerative disease, and in AD specifically, cannot be overstated. There is a rich clinical and pre-clinical literature base demonstrating alterations in cytokine levels in AD, including but not limited to, IL-1β, IL-6, TNFα, and interferon γ (INF-γ) (reviewed in [37]). What remains to be discerned is the meaning of the diverse array of activation phenotypes and how they contribute to, or modify, AD pathology. Therefore, neuroinflammation remains a prominent area of investigation and an attractive therapeutic target for AD treatment and/or prevention.

TNFα, a classic proinflammatory cytokine, innate immune system regulator and marker for neuroinflammation, has been shown to be dysregulated in AD. Clinical evidence suggests a central role for TNFα in AD pathogenesis with as much as a 25-fold elevation in TNFα in the cerebrospinal fluid of patients with AD [38] and findings that increased TNFα levels correlate with clinical deterioration [39]. However, a recent review of the literature from clinical examinations of TNFα in serum/plasma and CSF found considerable variability in TNFα regulation when comparing AD patients to cognitively normal controls [40]. This may reflect high variances in AD cohorts, especially in terms of AD progression. Perhaps of higher importance, generic assessment of serum/plasma and CSF compartments may not be reliable in contrast to direct assessment of isolated immune cells. In an effort to address this problem, a recent study used flow cytometry to detect TNFα in peripheral monocytes of cognitively normal, MCI and AD patients [41]. The study found a statistically significant difference in the percentage of monocytes producing TNFα between the controls and MCI and AD patients. MCI data fell in between normal controls and AD patients indicating that MCI is a prodromal stage of AD.

The pathophysiological role of TNFα in preclinical AD mouse models has been examined using a variety of approaches including genetic deletion of TNFα or TNF receptors (R) [34,42], biologic manipulation of TNFα levels [43] and other approaches (reviewed in [44]). Given the importance of TNFα signaling for immune function, abolishing TNFα signaling with genetic approaches makes data interpretation challenging and may also contribute to the varied findings with this approach. TNFRI deletion was shown to reduce APP processing and amyloid plaque formation [34] but TNFRI and RII ablation increased amyloid and tau pathology [42]. Deletion of TNFα reduced behavioral abnormalities in the PDAPP mouse model but increased late-stage amyloid burden [45]. Pharmacologic approaches with small molecules or biologics have shown some promise with thalidomide and infliximab reducing AD pathology [33,46]. However, there are limitations to some of these pharmacological approaches in that they require chronic injections and/or possess limiting risks and side effects [12,47–51]. Further, these studies have not made clear how much TNFα modulation is required for efficacy and what the chronic implications are for the innate immune system given the importance of TNFα in host defense and tumor growth control. The current data imply that a modest level of TNFα modulation, using an orally bioavailable small molecule, is effective in preventing cognitive decline and reducing PHF tau and insoluble amyloid pathology while not impairing the central or peripheral immune system or inducing other adverse effects.

Previous studies have shown that inflammation can induce BACE1 transcription and implicate the TNFα/TNFRI/NF-κB signaling pathway in BACE1 regulation [34,52]. Genetic deletion of TNFR1 [34], TNFRII [31] and treatment with high dose thalidomide [33] in the APP23 AD mouse model has been shown to reduce BACE1 protein levels and activity. In the current study, we did not observe a decrease in BACE1 protein with any dose of IDT although it is possible that BACE1 activity could be reduced by IDT treatment. However, it is unlikely that the primary effect of IDT is mediated through BACE1 as we did not see a significant reduction in soluble amyloid though there was a trend towards reduction in soluble Aβ40 and reduced BACE1 protein at the high IDT dose. Knocking out TNF signaling or more severe reductions in TNFα protein with high dose inhibitors may reduce amyloid pathology but at the expense of the immune system or cognitive function. Consistent with previous work [53], we hypothesize that with IDT treatment, rather than a direct effect on amyloid production, the microglia/macrophages may have an improved ability to clear amyloid pathology, particularly insoluble Aβ40.

We have previously demonstrated in the 3xTgAD mouse model that TNFα modulation using dithiothalidomide (DT), improved neuroinflammation, reduced leukocyte infiltration in the brain and improved cognition in younger mice exhibiting neuroinflammation but not yet developing significant amyloid or tau pathology [6]. Others have also demonstrated success with DT in older 3xTgAD mice as shown by improvement in memory function and reductions in the AD pathology markers phosphorylated tau and amyloid deposition [46], further validating TNFα as a drug target. In the current study, we have demonstrated success with a more effective and orally bioavailable TNFα modulator, IDT, in late-stage 3xTgAD mice with significant amyloid and tau pathology. 10 months of treatment with IDT in these mice was safe and the effective dose of 10 mg/kg is far lower than previous studies utilizing 50–56 mg/kg (i.p.) DT. Interestingly, we have noted differences in the results of TNFα modulation when the studies are conducted with younger versus older mice. In younger mice, TNFα modulation (using DT) was accompanied by a large reduction in infiltrating leukocyte populations and reductions in Iba1+ microglia numbers [6], however, we observed more subtle changes in leukocyte infiltration and Iba1+ microglia using IDT in older 3xTgAD mice. Although this could reflect a simple difference in the drug used, it is also feasible that the innate immune response is different as a result of age or advancing AD pathology. In support of this, human monocytes from AD patients challenged with LPS had a blunted cytokine response compared to both normal and MCI patients and may be the result of a persistent inflammatory state with age and disease progression that limits their capacity to respond [41]. Longitudinal studies of the innate immune response in normal aging and in neurodegeneration will inform treatment paradigms that may need to evolve over the course of a disease.

Despite a large body of literature emphasizing inflammation as a major component of AD pathology, we know very little about the precise changes in inflammatory status in innate immune cells and how these changes impact AD pathology and progression. Studies examining microglial phenotype and AD pathology in animal models reveal a complicated association. Published studies indicate that a strongly activated [10] macrophage phenotype is conducive to reducing amyloid accumulation [54] and anti-Aβ immunotherapy was shown to strongly induce IL-6 and TNFα just prior to a reduction in amyloid [55]. However, increases in amyloid burden have been noted in the presence of a mixed marker phenotype [8]. Our findings indicate a reduction in insoluble amyloid burden and PHF tau without the necessity to drastically shift macrophage/microglia phenotype or numbers.

Macrophages and microglia garner the most attention in examinations of neuroinflammation but the interplay of various innate immune cells is likely important. One understudied innate immune cell that is poised to influence AD pathology and progression is the neutrophil. Granulocytes, and subsequently neutrophilic granulocytes, arise from granulo-monocytic progenitors in the bone marrow and are a subset of white blood cells characterized by a polylobulated nucleus and cytoplasmic granules. Studies now suggest that the number and function of circulating neutrophils are reduced in AD patients [56,57] but whether this is a cause or consequence of the disease remains unresolved. High levels of TNFα are known to negatively regulate neutrophils by suppressing p40, a subunit of the IL-23 heterodimer. Combined with the p19 subunit, IL-23 promotes neutrophilia through regulation of granulopoiesis and IL-23 restores neutrophil numbers in p40 -/- mice [58]. Our current work demonstrates that IDT not only reduces TNFα levels, but also positively regulates p40 gene expression. Increased IL-23 is one potential mechanism for increased neutrophil infiltration in the brains of IDT-treated 3xTgAD mice. What remains to be determined is how these infiltrating neutrophils are functioning to promote a beneficial immune response and improved neuronal function.

**Fig 12:**
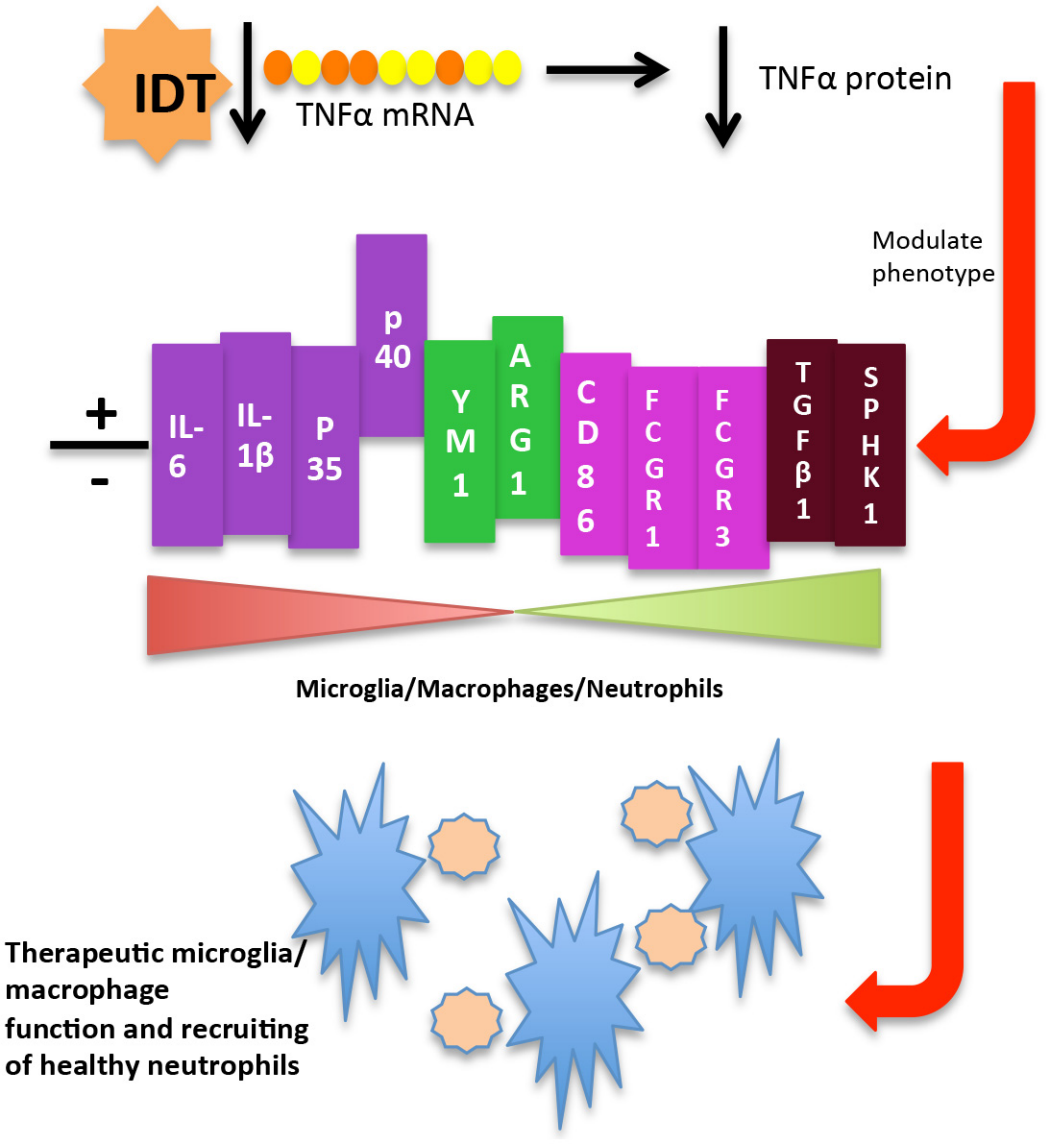
Schematic of IDT’s proposed mechanism of action. IDT destabilizes TNFα mRNA and reduces TNFα protein levels. Reduction of TNFα alters the expression of M1/M2 (N1/N2) genes and produces a novel mixed M1/M2 (N1/N2) phenotype. This may allow for improved microglia/macrophage (blue stars) function, recruiting of healthy neutrophils (beige circles) and improvement (+) of CNS reparative processes.

Neutrophils carry the stigma of a bad reputation when it comes to inflammation. The role played by neutrophils has long been viewed as restricted to the acute phase of inflammation and to resistance against extracellular pathogens [59]. Neutrophils are called into inflammatory sites by chemoattractant factors creating a potential role for neutrophils in the pathophysiology of AD because they are recruited into the brain by inflammation and components of amyloid plaques are inducers of some of their functions. Despite the considerable evidence for negative consequences associated with neutrophils such as neuron toxicity from cell-cell contact with neutrophils [60], there remains a considerable knowledge gap about the full spectrum of neutrophil activity in the brain under both normal and disease states. The cancer literature has provided evidence that neutrophils exhibit considerable plasticity in response to environmental signals [61] and are far more responsive to bidirectional cell-cell signaling that previously known. This indicates that we may have more control over shifting the response of innate immune cells to influence a therapeutic outcome than previously thought (summarized in Fig 12). Importantly, this change may be possible by modulating the peripheral immune system and the infiltration of innate immune cells, particularly neutrophils. There is a growing body of literature demonstrating that peripheral pharmacological treatments have beneficial effects on the brain [62]. Our results show good plasma levels of IDT following oral dosing yet there appears to be low brain exposure. It is conceivable that only a small amount of IDT is necessary for CNS activity. However, given the change in neutrophilia with IDT treatment, it is likely that peripheral leukocytes are directly affected and that infiltration kinetics into the CNS are subsequently modified.

In conclusion, our results indicate that modulation of TNFα using an orally available small molecule inhibitor reduces AD pathology, remodels the innate immune system and improves cognitive outcome in the 3xTgAD mouse model. Many questions remain to be answered about immune system function and the risk and progression of AD. Future studies will specifically address how cytokine modulation directly influences neutrophil and macrophage function and how this in turn influences AD pathology development and progression.

## Supporting Information

S1 Fig Stability of IDT in feed. TLC analysis showed the presence of IDT in Samples 2 (B; IDT feed immediately from the freezer) and 3 (C; IDT feed stored at rt for 7 days), but not in Sample 1 (A; Control diet). The HPLC trace for both the samples containing IDT (Samples 2 and 3) showed a peak at approximately 7 minutes (B and C), while this peak was absent in the trace for Sample 1 (A) lacking IDT. The peak seen at 7 minutes in A and B was confirmed to be that of IDT by spiking the samples with authentic IDT. Identical extraction procedures applied to equal quantities of feed (2.5 g) for Samples 1 and 2 resulted in the extraction of about 110 μg of IDT for Sample 2, and about 120 μg of IDT for Sample 3. No evidence of any decomposition products of IDT (containing the aromatic ring) was seen in any of the samples. These results confirm that IDT is stable in the feed for well over the 24 hour duration for which the animals are provided the feed.
(TIF)

S2 Fig Animal weights over IDT dosing period. Male (left) and female (right) mice were weighed weekly over the 38 week dosing period. Data represent mean ± SEM of n = 8–11/group.
(TIF)

S3 Fig Evaluation of splenic cell populations and TNFα. (A) The percentage of leukocytes (CD45Hi+), granulocytes (CD45Hi+/CD11b+/GR1+) and neutrophils (CD45Hi+/CD11b+/GR1+/1A8+) among all spleen cells analyzed across the four treatment groups. No significant differences in cell population were found. (B) Mean fluorescence intensity (MFI) of TNFα in leukocytes (CD45Hi+), granulocytes (CD45Hi+/CD11b+/GR1+) and neutrophils (CD45Hi+/CD11b+/GR1+/1A8+) and (C) The percentage of cells showing a positive TNFα signal was also analyzed in the same cell groups. No significant differences were found.
(TIF)

S4 Fig Serum Cytokine levels in IDT-treated mice. Serum collected at 16 months of age following 38 weeks of IDT treatment was analyzed for TNFα, IL-6, MCP-1 and CXCl-2. No significant differences were found by ANOVA between any of the cytokines evaluated but there was a strong trend for reduced TNFα and IL-6 in serum from the Low IDT group.
(TIF)
