# Supplementary figures and images for: Oral TNFα Modulation Alters Neutrophil Infiltration, Improves Cognition and Diminishes Tau and Amyloid Pathology in the 3xTgAD Mouse Model
Source: PLoS One. 2015 Oct 5;10(10):e0137305. doi: 10.1371/journal.pone.0137305 (PMC4593589; doi:10.1371/journal.pone.0137305)

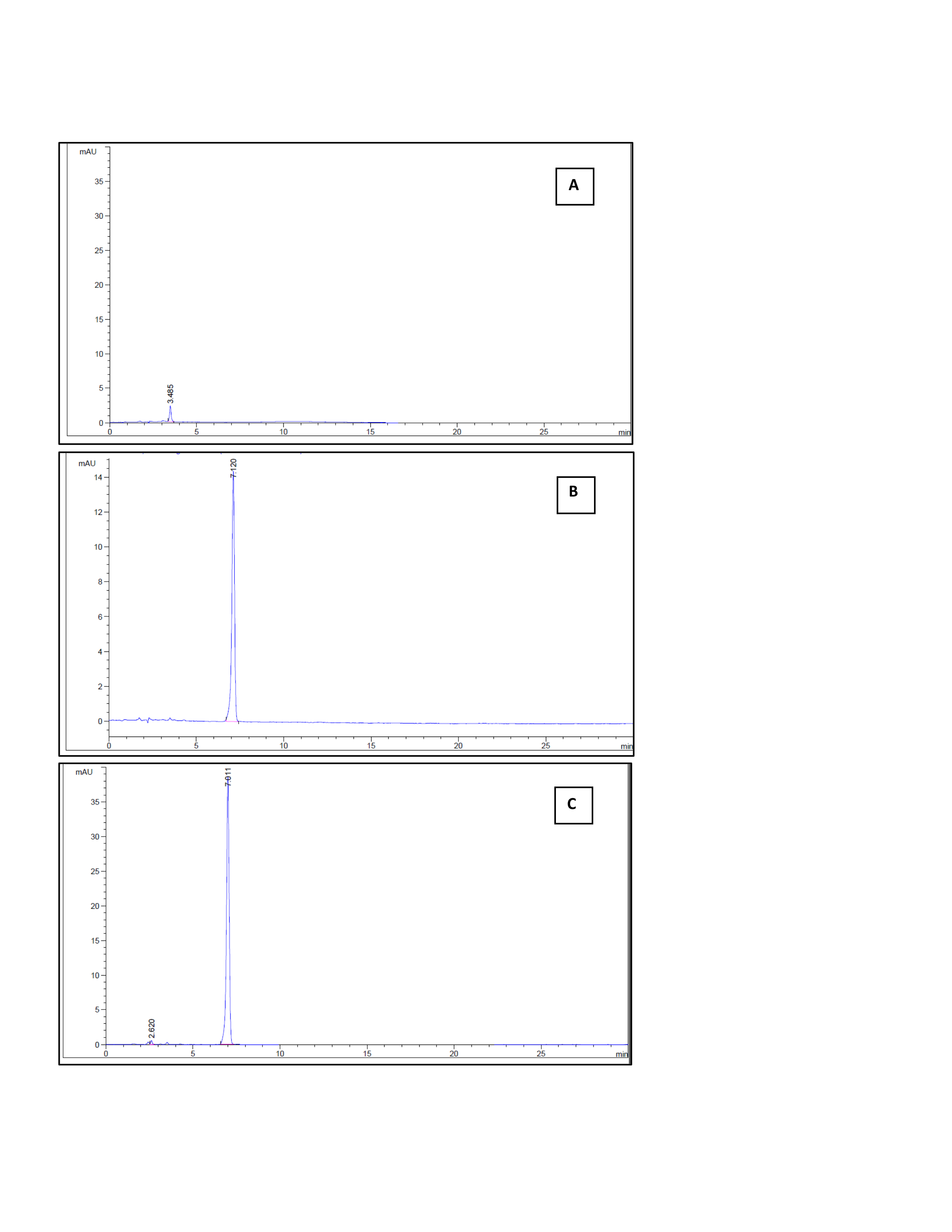

Supplement: S1 Fig — TLC analysis showed the presence of IDT in Samples 2 (B; IDT feed immediately from the freezer) and 3 (C; IDT feed stored at rt for 7 days), but not in Sample 1 (A; Control diet). The HPLC trace for both the samples containing IDT (Samples 2 and 3) showed a peak at approximately 7 minutes (B and C), while this peak was absent in the trace for Sample 1 (A) lacking IDT. The peak seen at 7 minutes in A and B was confirmed to be that of IDT by spiking the samples with authentic IDT. Identical extraction procedures applied to equal quantities of feed (2.5 g) for Samples 1 and 2 resulted in the extraction of about 110 μg of IDT for Sample 2, and about 120 μg of IDT for Sample 3. No evidence of any decomposition products of IDT (containing the aromatic ring) was seen in any of the samples. These results confirm that IDT is stable in the feed for well over the 24 hour duration for which the animals are provided the feed. (TIF) [file pone.0137305.s001.tif]

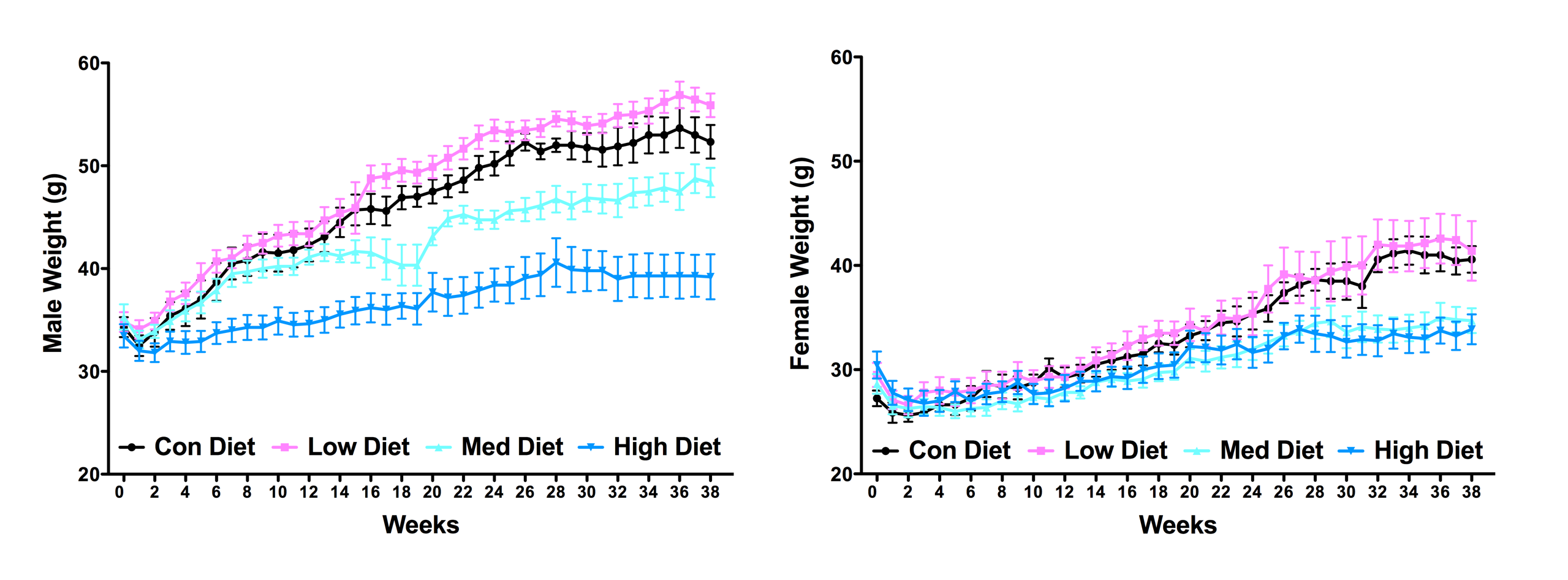

Supplement: S2 Fig — Male (left) and female (right) mice were weighed weekly over the 38 week dosing period. Data represent mean ± SEM of n = 8–11/group. (TIF) [file pone.0137305.s002.tif]

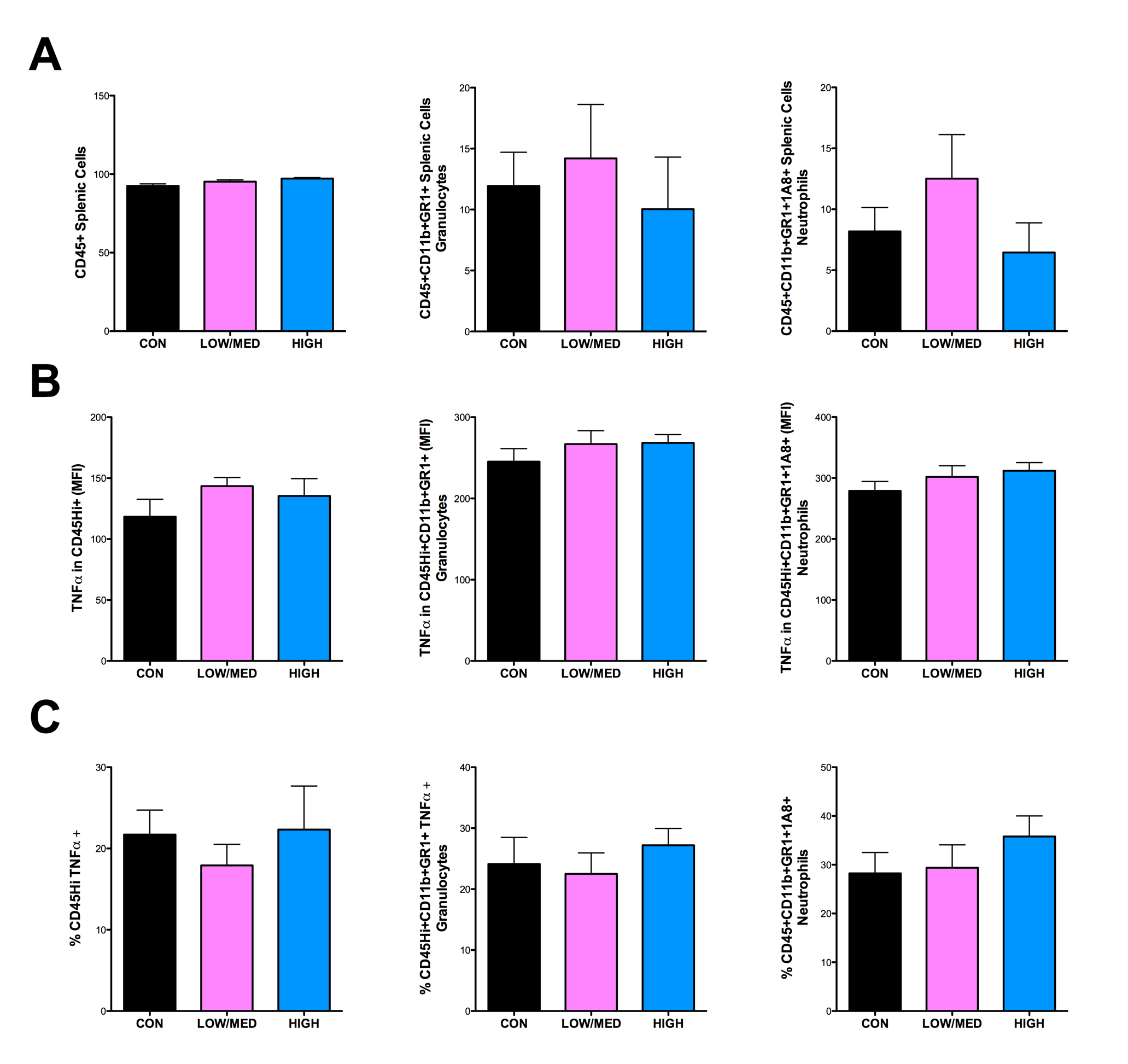

Supplement: S3 Fig — (A) The percentage of leukocytes (CD45Hi+), granulocytes (CD45Hi+/CD11b+/GR1+) and neutrophils (CD45Hi+/CD11b+/GR1+/1A8+) among all spleen cells analyzed across the four treatment groups. No significant differences in cell population were found. (B) Mean fluorescence intensity (MFI) of TNFα in leukocytes (CD45Hi+), granulocytes (CD45Hi+/CD11b+/GR1+) and neutrophils (CD45Hi+/CD11b+/GR1+/1A8+) and (C) The percentage of cells showing a positive TNFα signal was also analyzed in the same cell groups. No significant differences were found. (TIF) [file pone.0137305.s003.tif]

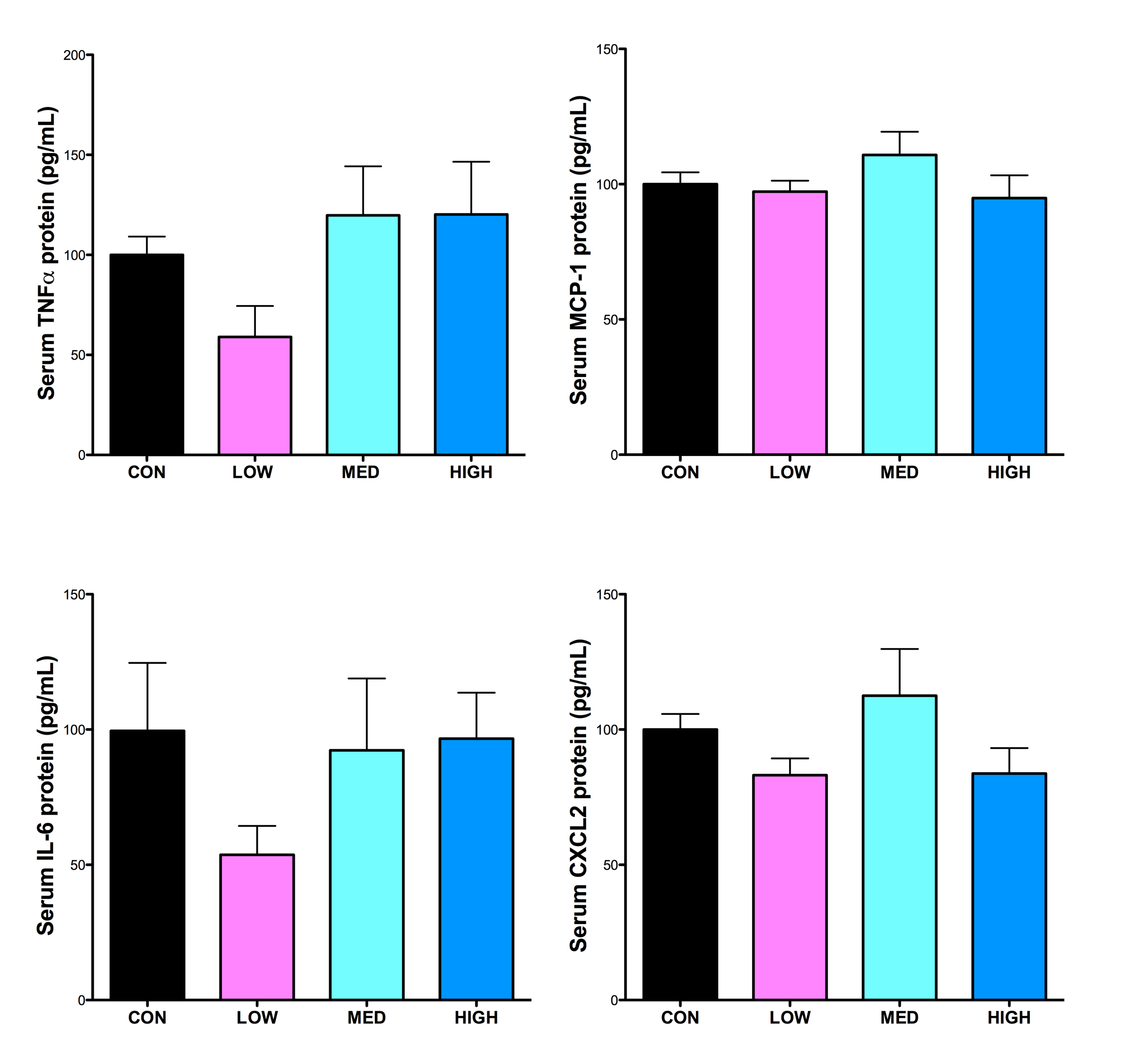

Supplement: S4 Fig — Serum collected at 16 months of age following 38 weeks of IDT treatment was analyzed for TNFα, IL-6, MCP-1 and CXCl-2. No significant differences were found by ANOVA between any of the cytokines evaluated but there was a strong trend for reduced TNFα and IL-6 in serum from the Low IDT group. (TIF) [file pone.0137305.s004.tif]
